# Expression and Functional Studies of Ubiquitin C-Terminal Hydrolase L1 Regulated Genes

**DOI:** 10.1371/journal.pone.0006764

**Published:** 2009-08-26

**Authors:** Anjali Bheda, Julia Shackelford, Joseph S. Pagano

**Affiliations:** 1 Lineberger Comprehensive Cancer Center, University of North Carolina, Chapel Hill, North Carolina, United States of America; 2 Departments of Medicine and Microbiology & Immunology, University of North Carolina, Chapel Hill, North Carolina, United States of America; 3 Department of Cell & Developmental Biology, University of North Carolina, Chapel Hill, North Carolina, United States of America; Johns Hopkins School of Medicine, United States of America

## Abstract

Deubiquitinating enzymes (DUBs) have been increasingly implicated in regulation of cellular processes, but a functional role for Ubiquitin C-terminal Hydrolases (UCHs), which has been largely relegated to processing of small ubiquitinated peptides, remains unexplored. One member of the UCH family, UCH L1, is expressed in a number of malignancies suggesting that this DUB might be involved in oncogenic processes, and increased expression and activity of UCH L1 have been detected in EBV-immortalized cell lines. Here we present an analysis of genes regulated by UCH L1 shown by microarray profiles obtained from cells in which expression of the gene was inhibited by RNAi. Microarray data were verified with subsequent real-time PCR analysis. We found that inhibition of UCH L1 activates genes that control apoptosis, cell cycle arrest and at the same time suppresses expression of genes involved in proliferation and migration pathways. These findings are complemented by biological assays for apoptosis, cell cycle progression and migration that support the data obtained from microarray analysis, and suggest that the multi-functional molecule UCH L1 plays a role in regulating principal pathways involved in oncogenesis.

## Introduction

Modification of proteins by ubiquitination is a fundamental mechanism for regulating numerous cellular processes including DNA repair, cell cycle regulation, antigen presentation, cell-cell communication, cell differentiation and apoptosis. Certain alterations in ubiquitination have been shown to lead to uncontrolled growth, finally leading to tumorigenesis [1]. Deubiquitination is a reversal of this process, carried out by deubiquitinating enzymes (DUBs), which are thiol proteases important for regulating different cellular processes [2]. Ubiquitin C-terminal hydrolase L1 (UCH L1) belongs to the family of DUBs [3] responsible for hydrolyzing carboxyl terminal esters and amides of ubiquitin. Additionally, it also possesses ubiquitin ligase activity [4] and functions as a mono-ubiquitin stabilizer [5]. This protein was isolated from the brain and at first considered a neuronal specific-marker [6]. Mutations in the *uch l1* gene are associated with neurodegenerative disorders such as Parkinson's, Huntington's and Alzheimer's diseases, but how these mutations relate to these diseases remains unclear [7].

More recently, UCH L1 has been detected in various types of malignant tissues [8]. UCH L1 levels are high in acute lymphoblastic leukemia cells [9], and in non-small cell lung cancer. UCH L1-positivity is associated with advanced stages of disease [10]. In addition, UCH L1 has been proposed as a key regulator of tumor invasion and metastasis [11]. Increased UCH L1 RNA levels are associated with poor prognosis in invasive breast cancer, and the protein has been suggested as a prognostic marker in ER/PR-negative tumors [12]. There is recent evidence that UCH L1 is highly expressed in pancreatic [13], prostate [14], medullary thyroid [15], esophageal [16] colorectal carcinomas [17], and in HPV16-transformed cells [18]. Additionally, UCH L1-positive renal cancer cells (RCC) had greater rates of proliferation and migration than UCH L1-negative RCC cells [19]. Finally, increased UCH L1 expression and activity were detected in Burkitt's lymphoma and Epstein-Barr Virus (EBV)-infected B-lymphocytes [20], and in these cell lines. UCH L1 is associated with enhanced proliferation and decreased cell adhesion properties [21].

This evidence suggests that UCH L1 may possess tumorigenic properties and promote tumor progression, although the mechanism is largely unknown. We wanted to investigate whether UCH L1 affects known oncogenic processes by utilizing the application of RNAi and cDNA microarray analyses to gain insight into genes regulated by UCH L1 in EBV-transformed B-cells and in SV40-transformed 293T HEK cells.

Our data demonstrate that suppression of UCH L1 causes alterations in the expression of genes related to cell death, migration, and cell cycle progression. To confirm the physiological consequences of such alterations, we assessed whether UCH L1 expression affects these pathways in biologic assays. Based on the results, we suggest that UCH L1 participates in oncogenesis by promoting proliferation and invasion, and by inhibiting apoptosis.

## Results and Discussion

### Identification of Genes Differentially Regulated by Suppression of UCH L1

**Figure 1:**
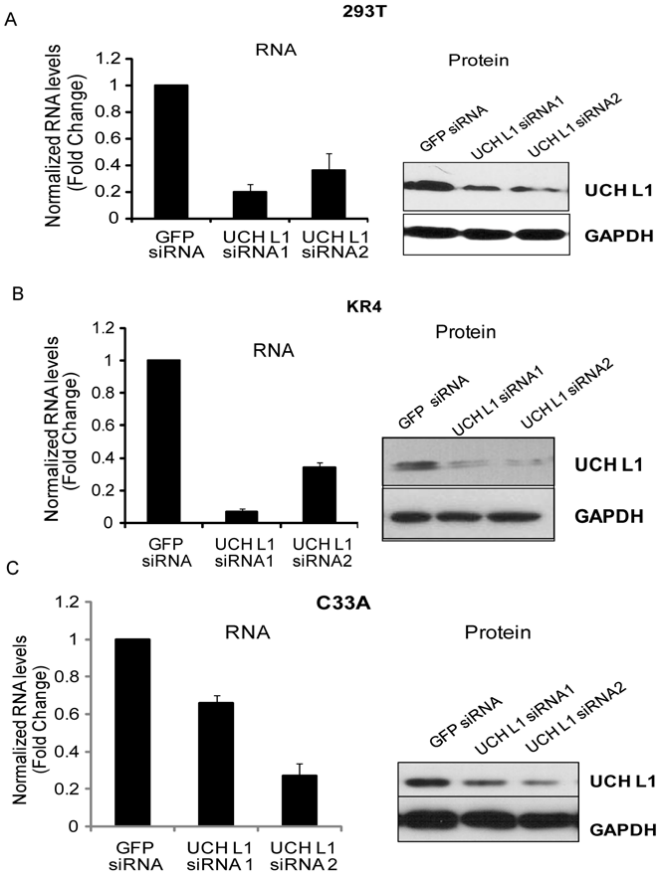
Suppression of UCH L1 expression by RNAi. HEK 293T or LCL KR4 cells were transfected with control GFP siRNA or either of two UCH L1 siRNAs in pRS vector and selected with puromycin. C33A cells were transiently transfected with control GFP siRNA or two UCH L1 siRNAs (for 48 h). Total RNA was extracted from control and UCH L1 siRNA-expressing cells and quantitative real-time PCR assays performed with gene-specific primers to determine the UCH L1 RNA levels. Each reaction was performed in triplicate and was normalized to GAPDH as an internal control. Whole-protein lysates were extracted from control and UCH L1 siRNA-expressing cells, and western blot analysis was performed for UCH L1 or GAPDH protein levels with specific antibodies. UCH L1 RNA and protein levels are shown for 293T (A) or KR4 (B) and C33A cells (C).

Microarray analysis to study the effects of UCH L1 on expression of cellular genes has been done in *gad* mice, in which the *uch l1* gene is deleted; however *gad* mice harbor additional mutations not specific to UCH L1 [22]. In addition, gene-expression profiles after over-expression of UCH L1 in an esophageal squamous carcinoma cell line indicated that expression led to induced expression of plaminogen activator inhibitor (PAI-1). PAI-1 is associated with cancer growth and metastasis [23]. To determine the alterations in global gene expression produced by suppression of UCH L1 levels, we used HEK 293T, SV40-transformed human embryonic kidney cells that grow as an adherent culture, and KR4, an EBV-transformed B-cell line that grows in suspension. These two cell types were chosen in order to identify a broad spectrum of specific gene-expression changes that could result from inhibition of UCH L1 expression. For the analyses, we created stable cell lines expressing GFP and two different UCH L1 siRNAs (UCH L1 siRNA1 and UCH L1 siRNA2) in both cell types. Total RNA extracted from the GFP and the two UCH L1 siRNA-expressing cell lines were used in microarray and QRT-PCR analyses. There was considerable reduction in UCH L1 RNA and protein levels in the 293T (**Figure 1A**) and KR4 cell lines (**Figure 1B**) expressing UCH L1 but not GFP siRNA. In addition, C33A, an HPV-negative cervical cancer cell line, was transiently transfected with GFP siRNAs and two different UCH L1 siRNA to compare the effects of UCH L1 suppression on a primary cancer cell line (**Figure 1C**). These results demonstrate that both the UCH L1 siRNAs were capable of effectively reducing UCH L1 RNA and protein levels by approximately 70% in 293T and KR4 cells. However, in C33A cells under transient transfection conditions (transfection efficiency ∼70–80%), suppression of UCH L1 was approximately 40%. To avoid off-target effects of siRNA, we employed 2 different siRNAs.

**Figure 2:**
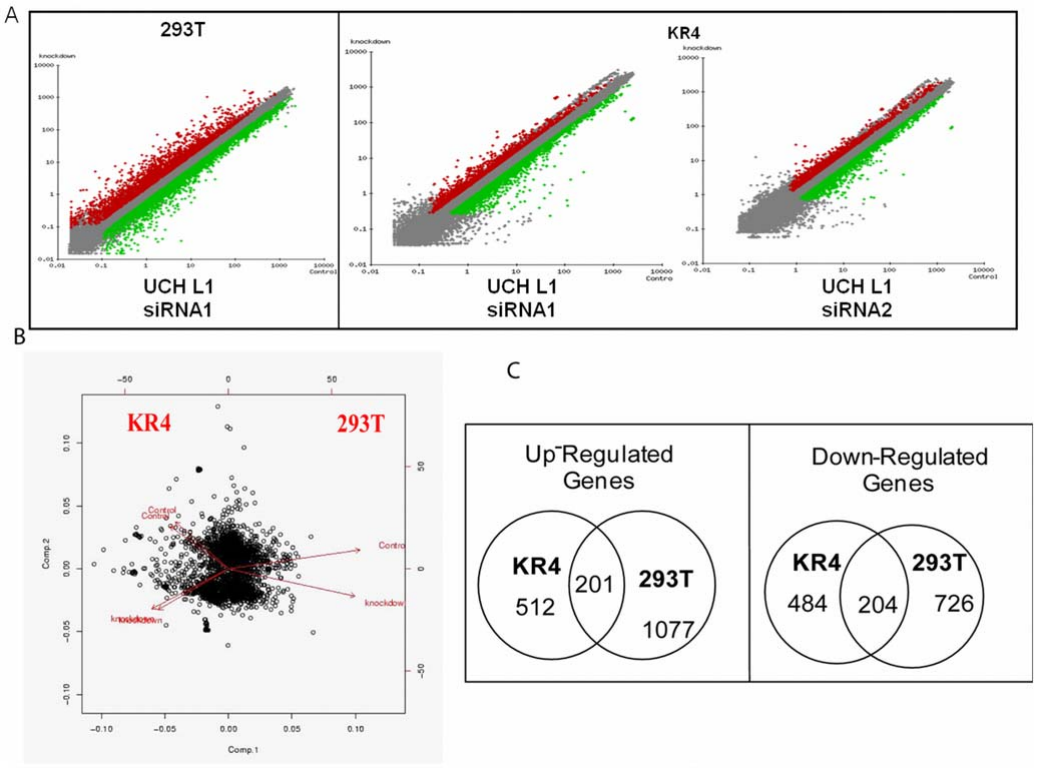
Identification of Statistically Significant Genes. Scatter-plots of signal intensity comparisons between GFP siRNA and UCH L1 siRNA. Each point represents a gene and the data: red points represent genes expressed to a higher degree in UCH L1 siRNA along the y-axis and green points represents genes expressed to a lower degree in UCH L1 siRNA along the x-axis. Grey squares represent genes changed less than 1.5 fold or with a p-value that failed to reach 0.05 (A). Unsupervised clustering of samples by principal component analysis (B). Each dot represents a gene on the plot. The distance between any pair of the samples is a function of relative similarity between the two samples. Venn diagram analyses (C) of gene list common in both cell types. The numbers in the center represent genes that are either up-regulated (left) or down-regulated genes (right) in common for both 293T and KR4 cells, whereas the numbers within the left circle represent genes regulated in KR4 independently of 293T and vice versa.

Gene-expression profiles were measured with RNA extracted from UCH L1 siRNA-expressing cells with the use of Agilent Whole Human Genome Array (44K) containing ∼41,000 unique probes. To strengthen the experimental design, we used UCH L1 siRNA1 and siRNA2 for KR4 and UCH L1 siRNA1 for 293T cells, respectively, to determine changes in gene expression. A dye-swap was performed for both cell types to eliminate dye-bias. Thus, the total number of RNA samples used for hybridization with 44K Agilent arrays was 12; altogether 6 arrays were used. The parameters used to identify significantly altered gene expression included: fold change of 1.5, Student's T test, normalization to the median, quality of 1 and log transformation of data. In addition, the Benjamini and Hochberg method of correction was applied to estimate the false discovery rate with a confidence of 5%. By applying these parameters we identified a number of statistically significant genes which were analyzed further for biological consequences of such changes. The quality of data generated was verified with scatter plots, which provide a graphical view of alterations in the control versus UCH L1 siRNA cell lines, for which the log of mean intensity for each pair of replicates was plotted (**Figure 2A**).

We next compared the mean values, signal intensities and distribution across the different samples. Box plots for control and UCH L1 siRNA samples demonstrate that the mean and range of signal amplitudes were very similar for all samples (data not shown). Principal Component Analysis (PCA) abridges the task of identifying sources of relative variability in a high dimensional data set by reducing the dimensions, and therefore the complexity, of the data set. PCA was used to determine the variability among the data set. As seen in **Figure 2B**, the UCH L1 siRNA samples differed from their respective controls and were clustered together when plotted according to correlation with the first two principal components. At the same time, the results also show that the two cell types are different.

Venn diagrams were used to identify the overlapping genes among different samples. We were interested in determining common genes regulated by UCH L1 in the two cell types, so we generated Venn diagrams by combining the separate gene lists obtained from Genesifter analysis after applying the selected parameters and then manually identified the common genes in the two cell types (**Figure 2C**). Of the total genes affected, 201 unique genes were up-regulated and 204 were down-regulated, which were common to both cell types. Table S1 (**Table S1**) shows the common gene list assembled from the affected genes. Among these, some genes were identified more than once as they were represented on the microarray by more than one probe set, which provided an additional internal control and increased confidence in the results. In addition, the Spike-In control showed slopes in the range of 0.997 to 0.88 across all the arrays, further increasing confidence in the data set.

### Validation of Microarray Data with Quantitative Real Time-PCR

**Figure 3:**
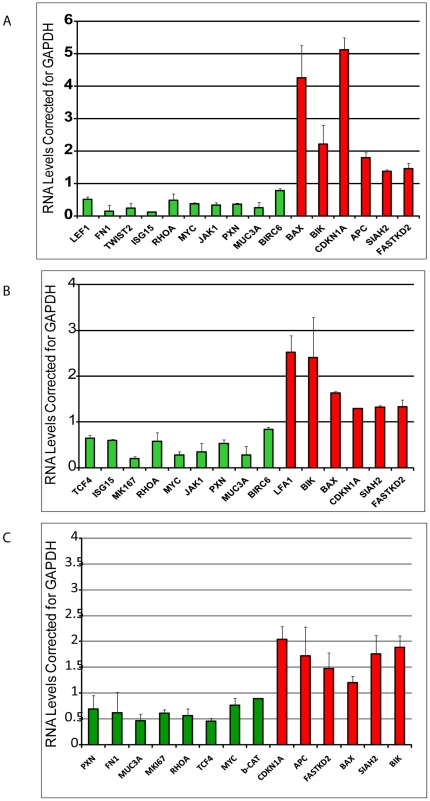
Validation of Selected Microarray Results by QRT-PCR. The same total RNAs isolated from GFP and the 2 UCH L1 siRNA-expressing cells as used in the microarray experiments were reverse-transcribed and quantitative real-time PCR was performed with gene-specific primers. Each reaction was performed in triplicate and normalized to their respective GFP siRNA control and also to GAPDH. QRT-PCR analysis for up- and down-regulated genes for 293T (A) and KR4 (B) and C33A cells (C).

To validate microarray results, QRT-PCR analysis was performed for 19 genes encoding proteins with known function. Genes selected were affected in both cell types and represented different physiological pathways (proteins involved in cell cycle, apoptosis, proliferation, and migration) and different ranges of fold change. We selected a few genes whose expression was altered only in 293T or in KR4 cells to demonstrate the specificity of the microarray data. We validated microarray data with respect to direction of change of expression (up or down) for 293T (**Figure 3A**), KR4 (**Figure 3B**) and for C33A cells (**Figure 3C**). We were able to validate some genes detected in only one or either cell type by microarray. Since the suppression of UCH L1 was lower in C33A cells, the extent to which these genes were altered in this cell line was different as compared to the other two cell lines. A comparison between microarray data and QRT-PCR showed good correlation (**Table 1**). The magnitude of changes for most of the significantly altered genes was larger in QRT-PCR assays than in the microarray data, as is commonly observed. Quantitative differences between the QRT-PCR and microarray results were probably due to variations in efficiency of cDNA synthesis among samples, primer-dimer formations, mispriming, and lower efficiency at later cycles as amplification products compete for DNA polymerase. Also, the normalization method used for microarray and QRT-PCR assays was different, especially in the number of genes used for normalization for microarray versus QRT-PCR.

**Table 1:** Comparison of Microarray and QRT-PCR Data.

Down-regulated genes	293T Cells *	293T Cells #	KR4 LCL *	KR4 LCLs #
**LEF1**	−1.82	0.51	Not detected	No change
**TWIST**	−8.2	0.24	Not detected	No change
**FN1**	−2.74	0.14	Not detected	No change
**RHOA**	−1.62	0.47	Not detected	0.58
**JAK1**	−.1 64	0.33	−1.67	0.35
**ISG15**	−1.6	0.12	−2.1	0.6
**MYC**	−1.85	0.36	Not detected	0.28
**PXN**	−4.85	0.36	− 2.31	0.53
**TCF4**	Not detected	No change	−1.52	0.65
**MUC3A**	−2.88	0.25	−3.35	0.28
**BIRC6**	Not detected	0.78	−1.75	0.84
**MKI67**	Not detected	No change	−1.57	0.29
**Up-regulated genes**	**293T Cells ** *	**293T Cells #**	**KR4 LCL ** *	**KR4 LCLs #**
**BAX**	1.73	4.25	2.09	1.63
**BIK**	1.71	2.2	1.59	2.41
**APC**	1.58	1.79	Not detected	No change
**CDKN1A**	2.57	5.11	1.58	1.29
**SIAH2**	1.52	1.37	1.71	1.34
**FASTKD2**	1.67	1.45	1.57	1.34

* Microarray fold change, # Quantitative real time PCR fold change

### Inhibition of UCH L1 Expression Induces Cell-Cycle Arrest and Apoptosis

**Figure 4:**
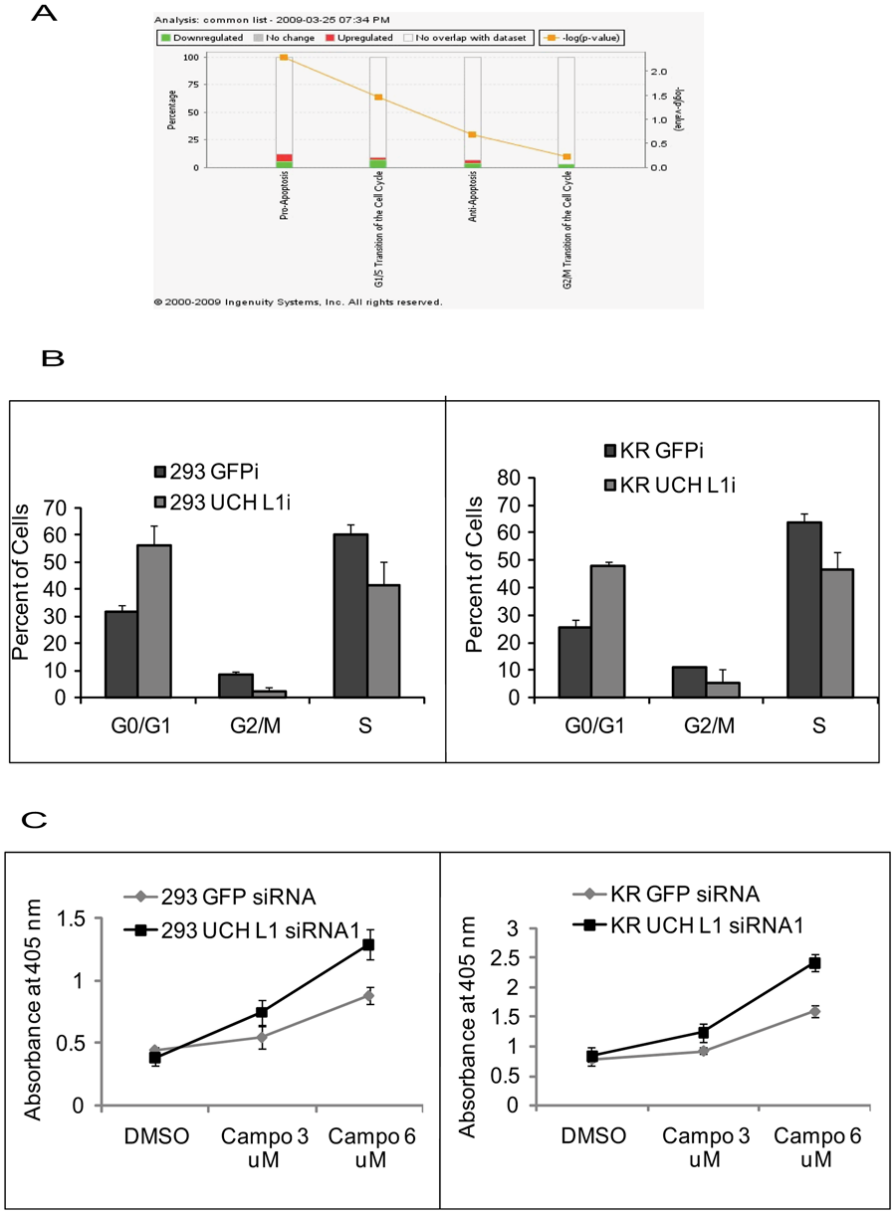
UCH L1 Suppression Induces G0/G1 Arrest and Apoptosis. Ingenuity pathway analysis was performed on genes in common between UCH L1 siRNA-expressing 293T and KR4 cell lines, after which tox list was generated for genes affecting apoptosis and cell-cycle (A). Cell-cycle analysis was performed on UCH L1 siRNA-expressing 293T (left panel) and KR4 (right panel) cells (B) after culturing the cells under sub-optimal reduced serum (1%) conditions for 24 h. For the analysis, 10^6^ cells were collected, fixed and stained with propidium iodide and then analyzed with flow cytometry. Representative histograms are shown in which cell count vs DNA content was plotted. Cell-cycle statistics were generated with ModFit software. To measure apoptosis, 293T and KR4 cells (10^4^) were treated with 3 and 6 µM concentrations of camptothecin for 10 h in (1%) serum. The number of viable cells was measured with a Cell Death ELISA assay, which measures the production of histone-associated DNA fragments at 405 nm wavelength. Each reaction was performed in duplicate. Graphical representation of cell death rate in 293T (left panel) and KR4 (right panel) cells (C).

According to the microarray analyses, inhibition of UCH L1 expression affected genes in multiple pathways, including transport, transcription, signal transduction, cytoskeleton, cell cycle, apoptosis, migration, and proliferation. Progression through cell cycle and inhibition of apoptosis are critical for oncogenesis. The microarray analysis indicated up-regulation of pro-apoptotic genes and down-regulation of genes involved in anti-apoptosis and cell cycle transition as seen in **Figure 4A**, which was generated after performing Ingenuity Pathway Analysis (IPA) on the common list of genes.

To test the role of UCH L1 in these pathways, we performed functional assays to elucidate the affected pathways. We first monitored whether UCH L1 suppression affected cell cycle progression in reduced serum (1%). The UCH L1 siRNA-expressing cell lines showed an accumulation of cells in the G0/G1 phase of the cell cycle, with a concomitant decrease in the proportion of those in S phase in sub-optimal reduced serum conditions (**Figure 4B**) for 293T and KR4 cells respectively. Similar results were obtained with both UCH L1 siRNAs (data not shown).

Since arrest in G0/G1 phase often leads to induction of apoptosis, and our data showed up-regulation of pro-apoptotic and down-regulation of anti-apoptotic genes, we tested whether UCH L1 siRNA-expressing cell lines were sensitive to induction of apoptosis. We performed a cell death assay in the presence or absence of the apoptosis-inducing agent, camptothecin, to determine cell death-rate by ELISA. The rate of induction of apoptosis by camptothecin was higher in UCH L1 siRNA-expressing cell lines as compared with control 293T and KR4 cells (**Figure 4C**). There was also an increase in DNA fragmentation in UCH L1 siRNA-expressing cell lines (**Figure S1**) treated with camptothecin, which confirms that the cells were in the last stage of apoptosis.

The results indicate that loss of UCH L1 expression by siRNA leads to induction of apoptosis due to arrest in the G0/G1 phase of cell-cycle. We are confident that the changes were specific to UCH L1 knockdown and not due to reduced serum levels, because EBV-transformed LCL are resistant to cell-cycle changes or apoptosis induction in reduced serum [24]. We did observe minor differences in cell-cycle profiles when cells were stimulated with 10% serum, but distinct changes in profiles were evident in sub-optimal reduced serum conditions. It has been reported that LDN-57444, an inhibitor of UCH L1's hydrolase activity, induced cell death via ER stress and also reduced cell viability in neuroblastoma cells [25]. Recently, UCH L1 was identified as an anti-apoptotic molecule when hepatoma cells were induced by UV radiation, this work supports our findings [26].

G1-S progression is regulated by the controlled expression and activity of various molecules such as Cyclins D, E and A, CDKs (CDK4, 6 and 2), CDKIs (INK4 and CIP/KIP family of proteins), Rb protein, and E2F transcription factors [27]. Our microarray data demonstrate the down-regulation of Cyclin G1, SerpinB9, BIRC6, and NAIP with simultaneous up-regulation of p21^WAF1^, CASP10, CARD9, BCCIP, CARD6, BAX, BIK, FASTKD2, and TNF-family member levels in both cell types.

Interestingly, we observed up-regulation of pRb1 and simultaneous down-regulation of the E2F2 gene in 293T cells, whereas in KR4 there was up-regulation of p53 indicating that probably the mechanism of action is different in the two cell types. Both p21^WAF1^ and p27^KIP1^ are well known tumor suppressors whose expression is decreased in cancer. The role of p21^WAF1^, a CDK inhibitor, in cell-cycle progression has been well established. BCCIP increases p21^WAF1^ expression and inhibits G1 to S progression [28]. In addition, we saw up-regulation of p27^KIP1^ in 293T cells; p27^KIP1^ causes arrest in G1 phase and induces apoptosis by increasing BAX protein levels [29]. Previous studies have demonstrated that down-regulation of UCH L1 expression in lung cancer cells leads to accumulation of p27^KIP1^, and an increased sensitivity to apoptosis. UCH L1 physically interacts with p27^KIP1^, which suggests a role for UCH L1 in p27^KIP1^ degradation [30], [31]. Our results indicate that loss of UCH L1 expression in both 293T and KR4 cells leads to G0/G1 arrest and apoptotic cell death. We believe that the mechanism of these effects is different in each case (**Figure 4**). These results suggest that in 293T cells G0/G1 cell-cycle arrest and apoptosis occur as a result of up-regulation of the Rb1 pathway, whereas in KR4 cells it occurs as a result of up-regulation of the p53 pathway.

### Reduced UCH L1 Expression Slows Cell Proliferation and Migration

**Figure 5:**
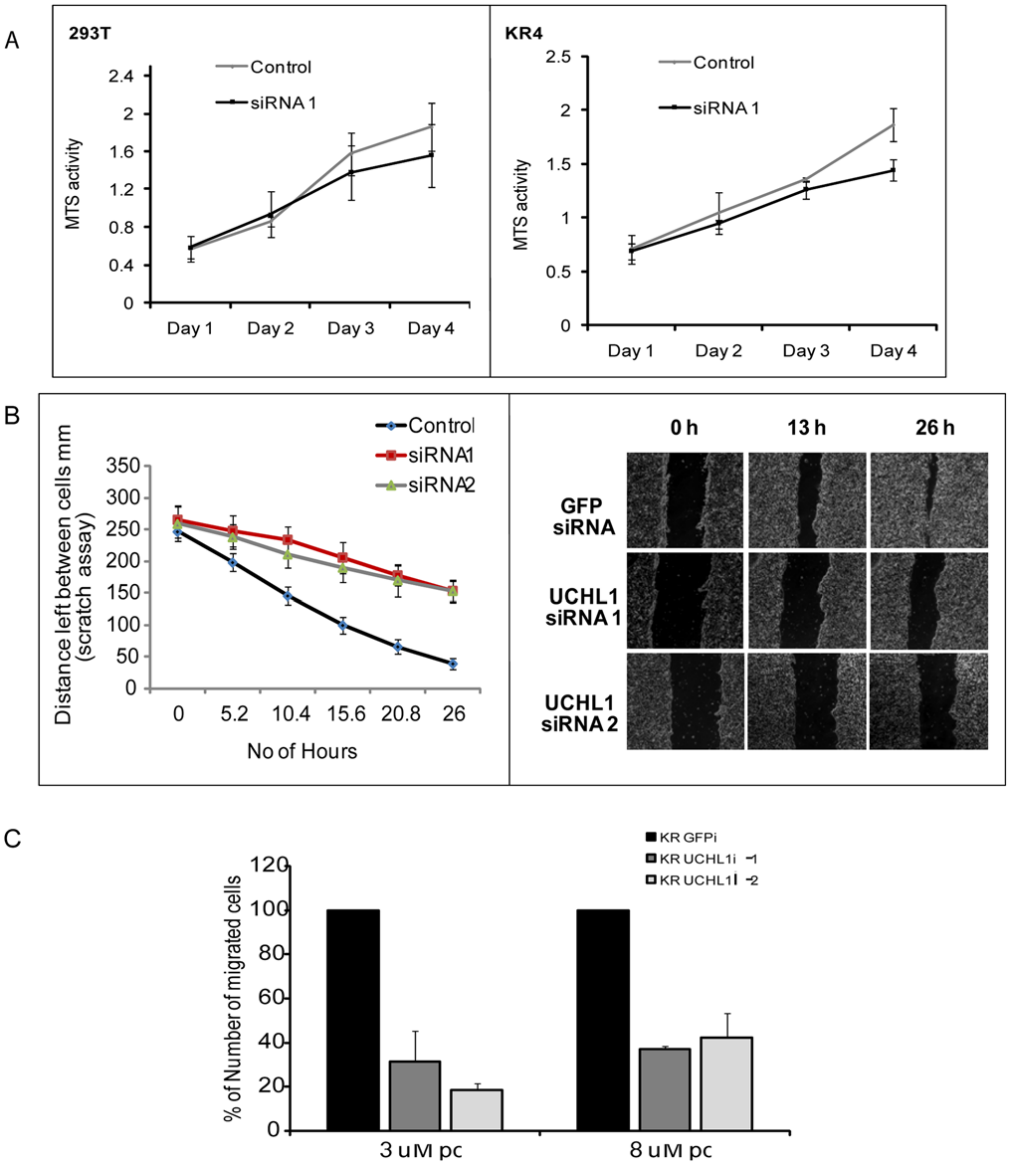
UCH L1 Suppression Inhibits Cell Proliferation and Migration. For cell proliferation assays, exact numbers of GFP or UCH L1 siRNA-expressing cells were cultured and cell proliferation rate was measured over a period of 4 days at absorbance at 490 nm for 293T (left panel) or KR (right panel) cells (A). Each reaction was performed in triplicate and data were calculated as a percentage of GFP siRNA. The proliferation rate of GFP siRNA cells was taken as 100%. Cell migration was assayed by wound healing for 293T cells and transmigration assay for KR4 cells. Cells were seeded on 6-well plates and grown to confluence in 10% serum. A scratch was introduced by scraping with a 10-µl pipette tip across the center of the monolayer. The cells were allowed to migrate and were monitored with Live Cell Imaging for 26 h. Images were collected every 30 m at 8 different locations for each control and UCH L1 siRNA-expressing 293T cell lines (B). Migration rate was calculated by measuring the distance between 2 points on the scratch for 6 different time points. For transwell migration, the same number of cells was seeded in the transwell (3 and 8-µM pore-size inserts) and the number of cells that migrated to the lower chamber were counted after 24 h (C). The data are expressed (wound assay and transmigration) as a percentage of GFP siRNA results, which was considered to be 100%.

Silencing of the *uch l1* gene is reported to reduce migration of H157 lung cancer cells[11]. Our results so far indicated that knockdown of UCH L1 leads to G0/G1 arrest along with induction of apoptosis. We next determined if loss of UCH L1 would also affect proliferative and migratory capacities of the cells. As seen in **Figure 5A**, MTS (3-(4,5-dimethylthiazol-2-yl)-5-(3-carboxymethoxyphenyl)-2-(4-sulfophenyl)-2H-tetrazolium) proliferation assays demonstrate slower growth rates in UCH L1 siRNA-expressing cell lines for both cell types and that the differences were only apparent on day 3 and day 4 of the assay. These results might be due to slowly cycling cells in the presence of UCH L1 siRNA. The delayed effect on proliferation rates might be because these assays were performed in optimal 10% serum conditions. We wanted to eliminate changes due to cell cycle arrest and increased cell death under reduced serum conditions, therefore the proliferation assays were performed in 10% serum.

We chose the *in-vitro* scratch assay for 293T adherent cells to examine their rate of migration by monitoring changes in distance between the cell boundaries in the scratched monolayers. Control and 2 different UCH L1 siRNA-expressing 293T cells (2×10^4^) were plated in each well of a 6-well plate in 10% serum and were allowed to grow until confluent before a wound was introduced into the cell monolayer. The migration of the cells was monitored for 26 h by Live Cell Imaging (**Figure 5B**). For lymphoid suspension cells, migration rate is measured with a transmigration assay. To examine the transmigration rate of KR4 cells, we plated 10^6^ GFP siRNA or UCH L1 siRNA-expressing cells in transwells with inserts of pore size 3 and 8 µM in a 12-well plate. Medium containing SDF-1, a B-cell specific factor, was used as a chemoattractant at a final concentration of 100 ng/ml. The migrated cells were counted 24 h post-seeding (**Figure 5C**). Changes in the rate of growth as well as a decrease in cell motility and migration of both 293T and KR4 cell lines were apparent with knockdown of UCH L1 expression. These data suggest that UCH L1 might be playing a role in cell migration and invasion. We are confident that the differences in migration rates are due to more slowly migrating cells, since the proliferation assay did not show significant differences in growth rate at day 2.

Genes involved in cell proliferation include cyclin D1, E2F, *c-myc*, DNA polymerase [32]. E2F family members play a major role during G1/S transition [33]. C-Myc is an oncogene involved in various cancers, and over-expression may be related to poor prognosis. C-Myc drives cell proliferation by up-regulating cyclins and down-regulating p21. The microarray data showed down-regulation of E2F, *c-myc*, and cyclin D1 gene expression and up-regulation of p21 in UCH L1 siRNA-expressing cells. The cellular oncogene, c-Myb, up-regulates the mouse UCH L1 promoter [34]. The microarray data showed down-regulation of Myb in UCH L1 siRNA-expressing cells 293T cells. This finding provided clues for further analysis of UCH L1 promoter sequence for the presence of putative binding sites for other factors. Interestingly, the UCH L1 promoter revealed c-Myc and E2F binding sites indicating that UCH L1 might be playing a role in proliferation via these molecules. The UCH L1 promoter also possessed binding sites for Twist2 and SMAD3, which were down-regulated, and LFA1, EGR1 and HNF4G, which were up-regulated in the microarray data in one of the two cell types. In addition, the UCH L1 promoter possesses binding sites for Lef1/TCF4 and the data demonstrate down-regulation of Lef1 in the 293T and TCF4 in the KR4 cell lines. Our data also demonstrate a positive feedback loop between regulation of UCH L1 and β-catenin indicating that UCH L1 affects this major oncogenic pathway [35] . These data encourage speculation that there may be correlation between genes identified by the microarray studies and the binding sites for specific gene products on the UCH L1 promoter.

The most lethal property of a malignant cell is its ability to metastasize. Adherent cells are bound to each another and to an extracellular matrix via cell adhesion molecules. In cancer cells, these molecules are either ablated or are compromised. Cell adhesion is essential for cell growth, migration and differentiation. The molecules that play roles in cell adhesion include cadherins, integrins, collagen, laminins, and cytoskeletal proteins. Important molecules involved in migration and invasion include fibronectin, paxillin, Rho, TIMPs, MMPs and many more [36]. The microarray data for 293T cells showed up-regulation of cadherins, integrins, actin, and myosin and at the same time down-regulation of vimentin, fibronectin and paxillin. Up-regulation of fibronectin and paxillin has been associated with malignant properties of cells [37], [38].

Recently, UCH L1 was shown to enhance the invasive capacity, cell adhesion and morphology of a lung cancer cell line via the AKT pathway, where phosphorylated AKT is reduced in the absence of UCH L1, although total AKT levels are not affected [11]. The phosphorylated form of AKT is the active form involved in tumorigenesis. The microarray data showed up-regulation of the *akt* gene in UCH L1 siRNA-expressing 293T cells, although we saw down-regulation of the phosphorylated AKT protein in these cells (**Figure S2**). These data indicate that UCH L1 might be mediating its effects on migration via the AKT pathway. Our microarray data also correlate with recently published data on the role of UCH L1 in B-cell proliferation and invasion [21]. Up-regulation of LFA-1 and its ligand ICAM was observed in UCH L1 siRNA-expressing KR4 cells. We also saw down-regulation of RhoA in UCH L1 siRNA-expressing 293T cells with the microarray and with QRT-PCR assays in KR4 cells. RhoA expression is increased in cancer and is required for migration and invasion of lymphoid tumor cells [39]. These data provide evidence that UCH L1 is involved in cell migration and invasion.

**Figure 6:**
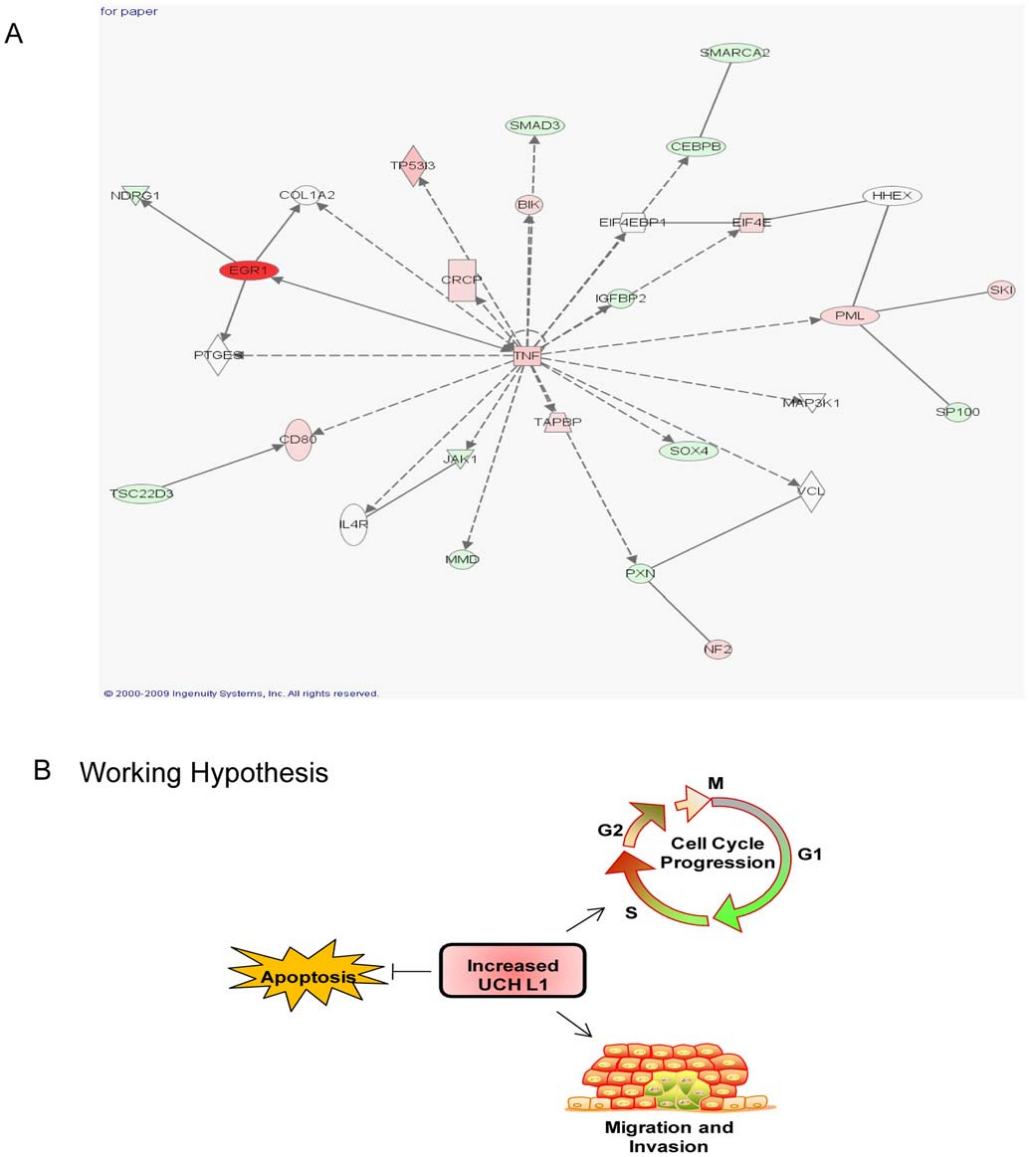
Ingenuity pathway analysis (IPA) network representation of the highly scored network. The genes that are shaded red are up-regulated and those that are green are down-regulated. The intensity of the color shows the degree of up-or down-regulation. A solid line represents a direct interaction while a dotted line represents an indirect interaction between the two gene products (A). Working Model: Increased expression of UCH L1 in malignant cells leads to cell cycle progression, increased proliferation and migration and at the same time abrogation of apoptotic pathways and immune responses and thus promotes tumor progression (B).

For further insight into genes regulated by UCH L1, we performed pathway analysis using the Ingenuity Pathway Analysis (IPA) [40], (www.ingenuity.com). Based on available data, this software created a highly ranked network (pathway model) from the list of genes we provided (**Figure 6A**). This network includes 29 genes whose products function in controlling cell death, cellular growth and proliferation and cell cycle. This information from IPA provides additional support for our results from physiological assays.

Taken together, the results of this study support the hypothesis that UCH L1 promotes tumor progression by inhibiting apoptosis and increasing cell proliferation and migration (**Figure 6B**). Exactly how UCH L1 regulates expression of these target genes will need further investigation. Besides being a deubiquitinating enzyme, UCH L1 has other activities [7] which are likely to play roles in other effects of this multi-functional protein.

## Materials and Methods

### Cell Culture

The human embryonic kidney 293T (HEK 293T) and C33A (HPV negative cervical cancer) cell lines ware cultured in DMEM (Sigma) supplemented with 10% FBS (Sigma) and penicillin–streptomycin. The lymphoblastoid cell line KR4 [41] was cultured in RPMI 1640 medium plus 10% heat-inactivated FBS and 100 units/ml penicillin–streptomycin. All cell lines were maintained at 37°C in 5% CO_2_ in air.

### Establishment of Stable UCH L1 siRNA Cells

UCH L1 siRNA and GFP siRNA was purchased from OriGene Technologies, Inc, in the form of an shRNA expression plasmid. The siRNAs used in the experiments are as follows: UCH L1 siRNA1: 5′ TGTGGCACAATCGGACTTATTCACGCAGT 3′; UCH L1 siRNA2: 5′ CCATGATGCCGTGGCACAGGAAGGCCAAT 3′. Stable cell lines were established as described in Supporting Information S1.

### Transient Transfections of UCH L1 siRNA

C33A cells were transiently transfected with UCH L1 siRNA1 and UCH L1 siRNA2 using Fugene HD (Roche) and RNA as well as protein were extracted 48 h post transfections.

### RNA Isolation, Probe Labeling and DNA Microarray

All experiments were performed according to the manufacturer's instructions. Total RNA was extracted with the use of Total RNA isolation mini kit (Agilent). The quality of RNA preparations was assessed on the Agilent 2100 Bioanalyzer with RNA 6000 Nano Reagents and RNA samples of RNA Integrity Number (RIN) 9.5 or above were used for all microarray experiments.

One microgram of total RNA (control and test) samples was amplified and labeled to generate cDNA for oligo microarrays with the Agilent low RNA input linear amplification kit (Cat. No 5184–3523). RNA Spike-Ins were used as internal control. The dye incorporation rate (between 1.4 and 1.6 pmol/µl) was measured with a Nanodrop® ND-1000 spectrophotometer (Agilent). Hybridization was performed using the Gene expression hybridization kit (Cat. No. 5188–5242) as described in Supporting Information S1.

### Statistical Data Analysis

The normalized data obtained from the Agilent Feature extraction software were pre-processed and all the internal control data points were eliminated. The data files were then analyzed with GeneSifter microarray data analysis software (VizX Labs). The parameters used to identify statistically significantly regulated genes were: Global normalization: median, Quality: 1, log transformation, threshold of 1.5-fold and Student's t-test with correction factor for false discovery rate (Benjamini and Hochberg). This software enables identification of differentially expressed genes and establishes biological significance based on Gene Ontology (GO) classification into biological process, molecular function and cellular component based on a z-score report. A z-score can be used to identify ontologies or pathway terms that are significantly over or under-expressed in a gene-list.

### Reverse Transcription and Real Time PCR (QRT PCR) Assay

Reverse transcription was performed with 500 ng total RNA (same as used in microarray experiments) using the iScript cDNA synthesis kit (Bio-Rad). A 1∶25 dilution of the cDNA reaction mixture was used in the QRT PCR reaction. QRT-PCR was carried out in a 15 µl reaction mixture with gene-specific primers with the iQ-SYBR green kit (Bio-Rad). PCR conditions: 95°C - 3 min, and 45 cycles of 95°C for 15 sec, 55°C for 45 sec on the ABI HT 7600 PCR instrument. All samples were assayed in triplicate. The differences in expression of gene-specific primers were evaluated using a relative quantification method in which the expression of specific gene was normalized to the reference gene GAPDH. The primers used are shown in (**Table S2)**.

### Immunoblot

Total cell lysates or immunocomplexes were resolved on 12% SDS-PAGE, transferred to PVDF membrane (GE Healthcare), blocked in 5% milk-Tris-buffered saline solution, and incubated at 4°C overnight with UCH L1 (1∶7500, Zymed), pAKT (Cell Signaling, 1∶1000), Total AKT (Cell Signaling, 1∶1000) and GAPDH (1∶5000, Sigma) antibodies followed with horse-radish peroxidase-conjugated secondary antibody. Proteins were detected with the Super Signal West Pico Chemiluminescence Detection Kit (Pierce Biotechnology, Rockford, IL, USA) and exposed to Kodak XAR-5 film.

### Transwell Migration Assay

1×10^5^ KR4 GFP or either of two UCH L1 siRNA cells were seeded in BD Biocoat 3-µm and 8-µm pore size control cell culture inserts (BD Biosciences) in RPMI-complete medium (10% FBS). The bottom chamber contained medium and 100 ng/ml SDF-1α chemoattractant. After an overnight incubation, cells that had migrated to the lower chamber of the transwell were collected and counted using a hemocytometer. The experiment was performed in duplicate.

### Proliferation MTS Assay

5,000 HEK 293T or 25,000 LCL KR4 cells were seeded in each well of 96-well plates for each GFP siRNA or two UCH L1 siRNA cells in a volume of 200 µl. CellTiter 96® Aq_ueous_ One Solution Cell Proliferation Assay (Promega) was used to determine the rate of proliferation following the manufacturer's instructions. MTS reagent 3-(4,5-Dimethylthiazol-2-yl)-5-(3-carboxymethoxyphenyl)-2-(4-sulfophenyl)-2H-tetrazolium was added from day 1 to 4 for 4 h and absorbance measured at 490 nm with a 550 plate-reader (Bio-Rad). The absorbance for each sample was subtracted from the blank and plotted.

### Live Cell Scratch Assay

HEK 293T stable UCH L1 or GFP siRNA-expressing cells were grown until they formed a monolayer in a 6-well plate in DMEM complete selection media. A wound was introduced in the monolayer of cells by scratching with a p10 pipette tip. Cells were washed twice with PBS to remove debris and given fresh media. The scratch was assigned time 0. Cells were allowed to proliferate and migrate into the wound during the next 26 h and images were collected for each cell type every 40 m, and migration of cells into the wound was recorded under a phase contrast view with Olympus IX70 inverted fluorescence microscope with a 4x phase objective.

### Apoptosis Assay by ELISA

Induction of apoptosis was measured by using the Cell Death ELISA Plus kit (Roche) to detect apoptotic nucleosomes. 10^4^ GFP or UCH L1 siRNA cells (HEK 293T or LCL KR4) were seeded in 96-well plates and each was treated either with DMSO or 3 or 6 µm concentrations of Camptothecin. After 16 h, histone-associated DNA fragments were quantified at 405 nm following the manufacturer's instructions. Each reaction was performed in duplicate.

### FACS Profile for Cell Cycle Analysis

For cell cycle analysis, cells were synchronized were seeded at 60% confluency and cultured for 24 h in reduced serum (1%). HEK 293T cells were trypsinized and harvested, washed once with cold phosphate-buffered saline, gently fixed with 80% cold ethanol, and incubated at 4°C for overnight. Fixed cells were centrifuged and resuspended in DNA-staining solution (1 mg/ml RNase A, 0.5 mg/ml propidium iodide), incubated at 37°C for 30 m and subjected to flow cytometry. DNA content was measured using Cytomation Summit software (Dako). Cell cycle was analyzed by ModFit LT software (Verity Software).

## Supporting Information

Supporting Information S1 Detailed materials and methods
(0.03 MB DOC)

Figure S1 Analysis of apoptotic DNA ladder. UCH L1 siRNA-expressing showed DNA-ladder formation after being cultured in reduced serum (1%) and 3 uM camptothecin for 10 h.
(1.32 MB TIF)

Figure S2 Western blot analysis showing role of AKT pathway. Western blot analysis was performed for pAKT, Total AKT, or GAPDH protein levels with specific antibodies on whole cell lysates extracted from 293T control and UCH L1 siRNA-expressing cells.
(0.80 MB TIF)

Table S1 List of genes regulated by UCH L1 commonly in 293T and KR4 cell lines
(0.59 MB DOC)

Table S2 List of QRT-PCR primers
(0.05 MB DOC)

## References

[1] Fuchs SY (2002). The role of ubiquitin-proteasome pathway in oncogenic signaling.. Cancer Biol Ther.

[2] Wilkinson KD (1997). Regulation of ubiquitin-dependent processes by deubiquitinating enzymes.. Faseb J.

[3] Wilkinson KD, Lee KM, Deshpande S, Duerksen-Hughes P, Boss JM (1989). The neuron-specific protein PGP 9.5 is a ubiquitin carboxyl-terminal hydrolase.. Science.

[4] Liu Y, Fallon L, Lashuel HA, Liu Z, Lansbury PT (2002). The UCH-L1 gene encodes two opposing enzymatic activities that affect alpha-synuclein degradation and Parkinson's disease susceptibility.. Cell.

[5] Osaka H, Wang YL, Takada K, Takizawa S, Setsuie R (2003). Ubiquitin carboxy-terminal hydrolase L1 binds to and stabilizes monoubiquitin in neuron.. Hum Mol Genet.

[6] Doran JF, Jackson P, Kynoch PA, Thompson RJ (1983). Isolation of PGP 9.5, a new human neurone-specific protein detected by high-resolution two-dimensional electrophoresis.. J Neurochem.

[7] Setsuie R, Wada K (2007). The functions of UCH-L1 and its relation to neurodegenerative diseases.. Neurochem Int.

[8] Campbell LK, Thomas JR, Lamps LW, Smoller BR, Folpe AL (2003). Protein gene product 9.5 (PGP 9.5) is not a specific marker of neural and nerve sheath tumors: an immunohistochemical study of 95 mesenchymal neoplasms.. Mod Pathol.

[9] Mohammad RM, Maki A, Pettit GR, al-Katib AM (1996). Bryostatin 1 induces ubiquitin COOH-terminal hydrolase in acute lymphoblastic leukemia cells.. Enzyme Protein.

[10] Sasaki H, Yukiue H, Moriyama S, Kobayashi Y, Nakashima Y (2001). Expression of the protein gene product 9.5, PGP9.5, is correlated with T-status in non-small cell lung cancer.. Jpn J Clin Oncol.

[11] Kim HJ, Kim YM, Lim S, Nam YK, Jeong J (2009). Ubiquitin C-terminal hydrolase-L1 is a key regulator of tumor cell invasion and metastasis.. Oncogene.

[12] Miyoshi Y, Nakayama S, Torikoshi Y, Tanaka S, Ishihara H (2006). High expression of ubiquitin carboxy-terminal hydrolase-L1 and -L3 mRNA predicts early recurrence in patients with invasive breast cancer.. Cancer Sci.

[13] Tezel E, Hibi K, Nagasaka T, Nakao A (2000). PGP9.5 as a prognostic factor in pancreatic cancer.. Clin Cancer Res.

[14] Leiblich A, Cross SS, Catto JW, Pesce G, Hamdy FC (2007). Human prostate cancer cells express neuroendocrine cell markers PGP 9.5 and chromogranin A.. Prostate.

[15] Takano T, Miyauchi A, Matsuzuka F, Yoshida H, Nakata Y (2004). PGP9.5 mRNA could contribute to the molecular-based diagnosis of medullary thyroid carcinoma.. Eur J Cancer.

[16] Takase T, Hibi K, Yamazaki T, Nakayama H, Taguchi M (2003). PGP9.5 overexpression in esophageal squamous cell carcinoma.. Hepatogastroenterology.

[17] Mizukami H, Shirahata A, Goto T, Sakata M, Saito M (2008). PGP9.5 methylation as a marker for metastatic colorectal cancer.. Anticancer Res.

[18] Wan F, Miao X, Quraishi I, Kennedy V, Creek KE (2008). Gene expression changes during HPV-mediated carcinogenesis: a comparison between an in vitro cell model and cervical cancer.. Int J Cancer.

[19] Seliger B, Fedorushchenko A, Brenner W, Ackermann A, Atkins D (2007). Ubiquitin COOH-terminal hydrolase 1: a biomarker of renal cell carcinoma associated with enhanced tumor cell proliferation and migration.. Clin Cancer Res.

[20] Rolen U, Kobzeva V, Gasparjan N, Ovaa H, Winberg G (2006). Activity profiling of deubiquitinating enzymes in cervical carcinoma biopsies and cell lines.. Mol Carcinog.

[21] Rolen U, Freda E, Xie J, Pfirmann T, Frisan T (2008). The Ubiquitin C-terminal Hydrolase UCH-L1 regulates B-cell proliferation and integrin activation.. J Cell Mol Med.

[22] Bonin M, Poths S, Osaka H, Wang YL, Wada K (2004). Microarray expression analysis of gad mice implicates involvement of Parkinson's disease associated UCH-L1 in multiple metabolic pathways.. Brain Res Mol Brain Res.

[23] Hibi K, Kodera Y, Ito K, Akiyama S, Shirane M (2004). Plasminogen activator inhibitor-1 is a downstream mediator of the PGP9.5-related oncogenic pathway in esophageal squamous cell carcinoma.. Anticancer Res.

[24] Spender LC, Cannell EJ, Hollyoake M, Wensing B, Gawn JM (1999). Control of cell cycle entry and apoptosis in B lymphocytes infected by Epstein-Barr virus.. J Virol.

[25] Tan YY, Zhou HY, Wang ZQ, Chen SD (2008). Endoplasmic reticulum stress contributes to the cell death induced by UCH-L1 inhibitor.. Mol Cell Biochem.

[26] Hsieh SY, Hsu CY, He JR, Liu CL, Lo SJ (2009). Identifying Apoptosis-Evasion Proteins/Pathways in Human Hepatoma Cells via Induction of Cellular Hormesis by UV Irradiation.. J Proteome Res.

[27] Massague J (2004). G1 cell-cycle control and cancer.. Nature.

[28] Meng X, Liu J, Shen Z (2004). Inhibition of G1 to S cell cycle progression by BCCIP beta.. Cell Cycle.

[29] Maddika S, Ande SR, Panigrahi S, Paranjothy T, Weglarczyk K (2007). Cell survival, cell death and cell cycle pathways are interconnected: implications for cancer therapy.. Drug Resist Updat.

[30] Shen H, Sikorska M, Leblanc J, Walker PR, Liu QY (2006). Oxidative stress regulated expression of ubiquitin Carboxyl-terminal Hydrolase-L1: role in cell survival.. Apoptosis.

[31] Caballero OL, Resto V, Patturajan M, Meerzaman D, Guo MZ (2002). Interaction and colocalization of PGP9.5 with JAB1 and p27(Kip1).. Oncogene.

[32] Schmidt EV (1999). The role of c-myc in cellular growth control.. Oncogene.

[33] Sun A, Bagella L, Tutton S, Romano G, Giordano A (2007). From G0 to S phase: a view of the roles played by the retinoblastoma (Rb) family members in the Rb-E2F pathway.. J Cell Biochem.

[34] Long EM, Long MA, Tsirigotis M, Gray DA (2003). Stimulation of the murine Uchl1 gene promoter by the B-Myb transcription factor.. Lung Cancer.

[35] Bheda A, Yue W, Gullapalli A, Whitehurst C, Liu R (2009). Positive reciprocal regulation of ubiquitin C-terminal hydrolase L1 and beta-catenin/TCF signaling.. PLoS One.

[36] Cairns RA, Khokha R, Hill RP (2003). Molecular mechanisms of tumor invasion and metastasis: an integrated view.. Curr Mol Med.

[37] Labat-Robert J (2002). Fibronectin in malignancy.. Semin Cancer Biol.

[38] Sattler M, Pisick E, Morrison PT, Salgia R (2000). Role of the cytoskeletal protein paxillin in oncogenesis.. Crit Rev Oncog.

[39] Yan B, Chour HH, Peh BK, Lim C, Salto-Tellez M (2006). RhoA protein expression correlates positively with degree of malignancy in astrocytomas.. Neurosci Lett.

[40] Mayburd AL, Martlinez A, Sackett D, Liu H, Shih J (2006). Ingenuity network-assisted transcription profiling: Identification of a new pharmacologic mechanism for MK886.. Clin Cancer Res.

[41] Kozbor D, Lagarde AE, Roder JC (1982). Human hybridomas constructed with antigen-specific Epstein-Barr virus-transformed cell lines.. Proc Natl Acad Sci U S A.

