# Supplementary material for: Expression and Functional Studies of Ubiquitin C-Terminal Hydrolase L1 Regulated Genes
Source: PLoS One. 2009 Aug 26;4(8):e6764. doi: 10.1371/journal.pone.0006764 (PMC2729380; doi:10.1371/journal.pone.0006764)
Supplement: Supporting Information S1 — Detailed materials and methods (0.03 MB DOC) [file pone.0006764.s001.doc]

**Supplementary Information**

**Supplementary Materials and Methods:**

**Establishment of Stable UCH L1 siRNA Cells.** For the establishment of UCH L1 siRNA and control siRNA stable lines, HEK 293T cells were transfected with 2 μg of respective plasmid with the use of Fugene HD (Roche Diagnostics). Cells were passaged 24 h post transfections and selection was started at 48 h. 2 x 10^4^ LCL KR4 cells were nucleofected (Amaxa technologies) with 4 μg after respective plasmid, allowed to recover and selection with puromycin was started 48 h post nucleofection. All cells were maintained in the selective media contained 2µg/ml puromycin (InvivoGen).

**DNA Microarray Hybridization.** 750 ng UCH L1 siRNA cRNA was mixed with 750 ng GFP siRNA cRNA in the presence of target controls, fragmentated by heating (30 min at 60°C) and then hybridized on 44K Human Whole-Genome 60-mer microarrays (G4112A, Agilent) in a rotary oven (4000 rpm, 60°C, 17 h). Slides were disassembled and washed in solutions I and II and dried using a nitrogen-filled air gun before scanning. Microarrays were scanned with a Dual laser DNA microarray scanner (Agilent) were red and green PMTs were each set at 100%, and scan resolution was set to 10 μm. Images were analyzed and data was extracted, background subtracted and normalized using the standard procedures of Feature Extraction Software A.7.5.1 (Agilent). All samples had technical replicates, and each replicate was run on separate slides to eliminate any dye bias.

**Isolation and Detection of DNA Fragmentation.** For isolation of DNA, 5 x 10^6^ cells treated with campothecin (3 μM) were lysed in the lysis buffer containing 0.5 % Triton 100X, 5 mM Tris ph 7.4, 20 mM EDTA, 250 mM NaCL, and 20 mg/ml proteinase K. The cells were incubated on ice for 20 m and then at 56 C. The DNA was then isolated by phenol chloroform extraction followed by ethanol precipitation. The precipitated DNA was dissolved in TE buffer containing 20 mg/ml RNase A at 37 C. The samples were resolved on a 1.5 % TAE agarose gel (MP agarose, Boehringer—Mannheim) at 40V for 2 h and visualized by UV light after standard ethidium bromide staining.
