# Supplementary figures and images for: Expression and Functional Studies of Ubiquitin C-Terminal Hydrolase L1 Regulated Genes
Source: PLoS One. 2009 Aug 26;4(8):e6764. doi: 10.1371/journal.pone.0006764 (PMC2729380; doi:10.1371/journal.pone.0006764)

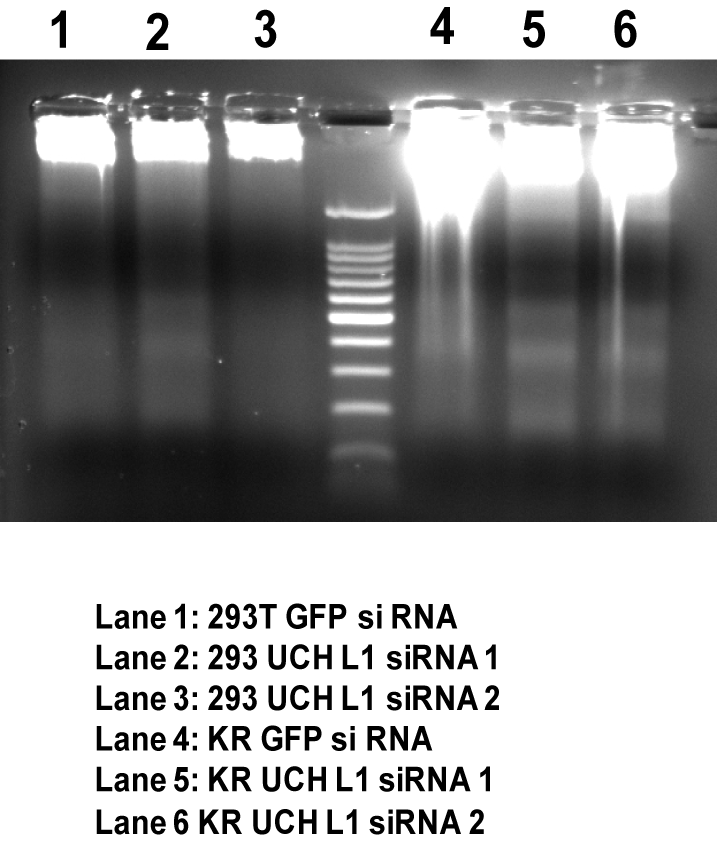

Supplement: Figure S1 — Analysis of apoptotic DNA ladder. UCH L1 siRNA-expressing showed DNA-ladder formation after being cultured in reduced serum (1%) and 3 uM camptothecin for 10 h. (1.32 MB TIF) [file pone.0006764.s002.tif]

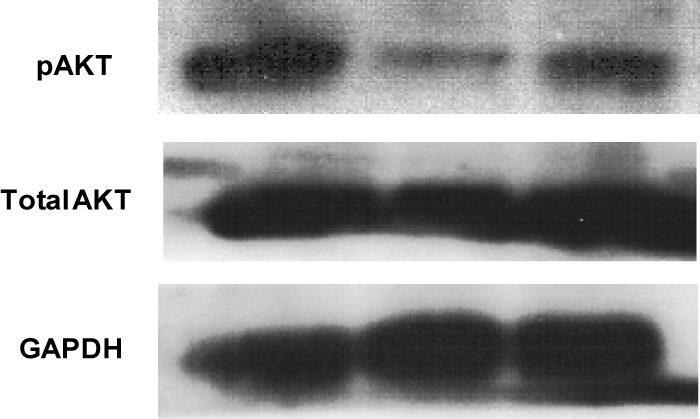

Supplement: Figure S2 — Western blot analysis showing role of AKT pathway. Western blot analysis was performed for pAKT, Total AKT, or GAPDH protein levels with specific antibodies on whole cell lysates extracted from 293T control and UCH L1 siRNA-expressing cells. (0.80 MB TIF) [file pone.0006764.s003.tif]
