# Supplementary material for: Expression and Functional Studies of Ubiquitin C-Terminal Hydrolase L1 Regulated Genes
Source: PLoS One. 2009 Aug 26;4(8):e6764. doi: 10.1371/journal.pone.0006764 (PMC2729380; doi:10.1371/journal.pone.0006764)
Supplement: Table S1 — List of genes regulated by UCH L1 commonly in 293T and KR4 cell lines (0.59 MB DOC) [file pone.0006764.s004.doc]

| **Gene ID** | **Gene Name** | **Fold Change KR4 Cells** | **Fold Change 293T Cells** | **Direction** | **Accession Number** |
| --- | --- | --- | --- | --- | --- |
| 7-Mar | Membrane-associated ring finger (C3HC4) 7 | 1.5 | 2.65 | Down | NM_022826 |
| ADSSL1 | Adenylosuccinate synthase like 1 | 1.65 | 2.22 | Down | NM_199165 |
| AGTPBP1 | ATP/GTP binding protein 1 | 1.68 | 1.69 | Down | NM_015239 |
| ALDH2 | Aldehyde dehydrogenase 2 family (mitochondrial) | 1.59 | 1.6 | Down | NM_000690 |
| ALPK1 | Alpha-kinase 1 | 2.13 | 4.13 | Down | AK026323 |
| ANKFY1 | Ankyrin repeat and FYVE domain containing 1 | 1.87 | 4.31 | Down | NM_020740 |
| ANKK1 | Ankyrin repeat and kinase domain containing 1 | 1.99 | 4.77 | Down | NM_178510 |
| ANKRD10 | Ankyrin repeat domain 10 | 1.91 | 3.5 | Down | NM_017664 |
| ANKS6 | Ankyrin repeat and sterile alpha motif domain containing 6 | 1.54 | 1.72 | Down | NM_173551 |
| AP1S2 | Adaptor-related protein complex 1, sigma 2 subunit | 2 | 1.96 | Down | NM_003916 |
| ARHGAP24 | Rho GTPase activating protein 24 | 6.14 | 2.2 | Down | AK130576 |
| ARHGEF10 | Rho guanine nucleotide exchange factor (GEF) 10 | 3.34 | 6.07 | Down | NM_014629 |
| ARID2 | AT rich interactive domain 2 (ARID, RFX-like) | 2.18 | 2.44 | Down | AK055181 |
| ARL3 | ADP-ribosylation factor-like 3 | 2.06 | 1.58 | Down | NM_004311 |
| ATXN2L | Ataxin 2-like | 1.57 | 1.6 | Down | NM_145714 |
| BBS5 | Bardet-Biedl syndrome 5 | 1.79 | 2.63 | Down | NM_152384 |
| BCAR1 | Breast cancer anti-estrogen resistance 1 | 1.57 | 2.3 | Down | AK127004 |
| BCGF1 | B-cell growth factor | 2.24 | 4.67 | Down | M15530 |
| BCR | Breakpoint cluster region | 1.69 | 1.67 | Down | NM_004327 |
| BTBD14B | BTB (POZ) domain containing 14B | 1.84 | 2.74 | Down | NM_052876 |
| CAPRIN2 | Caprin family member 2 | 1.5 | 1.72 | Down | NM_001002259 |
| CATSPER2 | Cation channel, sperm associated 2 | 1.6 | 2.87 | Down | NM_172097 |
| CCDC144B | Coiled-coil domain containing 144A | 1.83 | 4.34 | Down | NM_182568 |
| CCDC74B | Coiled-coil domain containing 74B | 2.64 | 1.63 | Down | NM_207310 |
| CDT1 | Chromatin licensing and DNA replication factor 1 | 1.67 | 1.71 | Down | NM_030928 |
| CEBPB | CCAAT/enhancer binding protein (C/EBP), beta | 1.72 | 2.13 | Down | AI431972 |
| CECR4 | Similar to hCG1804364 | 2.23 | 2.87 | Down | AF307448 |
| CENPH | Centromere protein H | 1.67 | 1.57 | Down | NM_022909 |
| CHRD | Chordin | 2.26 | 2.85 | Down | NM_003741 |
| CHST6 | Carbohydrate (N-acetylglucosamine 6-O) sulfotransferase 6 | 3.53 | 1.89 | Down |  |
| CIT | Citron (rho-interacting, serine/threonine kinase 21) | 1.93 | 1.79 | Down | NM_007174 |
| CLIC4 | Chloride intracellular channel 4 | 1.67 | 1.53 | Down | NM_013943 |
| CLTC | Clathrin, heavy chain (Hc) | 1.51 | 2.11 | Down | AF130062 |
| CMTM7 | CKLF-like MARVEL transmembrane domain containing 7 | 1.63 | 2.16 | Down | NM_138410 |
| CNOT4 | CCR4-NOT transcription complex, subunit 4 | 1.64 | 1.85 | Down | BC035590 |
| CNOT6 | CCR4-NOT transcription complex, subunit 6 | 1.69 | 1.62 | Down | NM_015455 |
| CPN2 | Carboxypeptidase N, polypeptide 2 | 2.85 | 6.51 | Down | BC042334 |
| CROCCL2 | Ciliary rootlet coiled-coil, rootletin-like 2 | 73.91 | 31.99 | Down | BC033082 |
| CROP | Cisplatin resistance-associated overexpressed protein | 1.58 | 4.1 | Down | NM_016424 |
| CSH1 | Chorionic somatomammotropin hormone 1 (placental lactogen) | 5.3 | 1.65 | Down | NM_022640 |
| CTDSP2 | CTD (carboxy-terminal domain, RNA polymerase II, polypeptide A) small phosphatase 2 | 2 | 1.76 | Down | NM_005730 |
| CTSL2 | Cathepsin L2 | 3.38 | 1.52 | Down | NM_001333 |
| CYP1A2 | Cytochrome P450, family 1, subfamily A, polypeptide 2 | 2.02 | 3.19 | Down | NM_000761 |
| DENND1B | DENN/MADD domain containing 1B | 2.65 | 2.31 | Down | AL831839 |
| DENND4A | DENN/MADD domain containing 4A | 1.52 | 2.21 | Down | NM_005848 |
| DHRS2 | Dehydrogenase/reductase (SDR family) member 2 | 4.92 | 1.7 | Down | NM_182908 |
| DNAH17 | Dynein, axonemal, heavy chain 17 | 1.52 | 8.93 | Down | AK090407 |
| DOCK11 | Dedicator of cytokinesis 11 | 1.71 | 2.11 | Down | NM_144658 |
| DUB3 | Deubiquitinating enzyme 3 | 1.8 | 3.25 | Down | NM_201402 |
| EEF1A1 | Eukaryotic translation elongation factor 1 alpha 1 | 1.61 | 1.76 | Down | NM_001402 |
| EIF1B | Eukaryotic translation initiation factor 1B | 1.51 | 1.76 | Down | NM_005875 |
| EMR1 | Egf-like module containing, mucin-like, hormone receptor-like 1 | 3.5 | 1.79 | Down | NM_001974 |
| ENO3 | Enolase 3 (beta, muscle) | 1.52 | 2.08 | Down | NM_001976 |
| FBLN5 | Fibulin 5 | 1.95 | 1.88 | Down | NM_006329 |
| FYN | FYN oncogene related to SRC, FGR, YES | 2.36 | 1.52 | Down | NM_002037 |
| GALNT3 | UDP-N-acetyl-alpha-D-galactosamine:polypeptide N-acetylgalactosaminyltransferase 3 (GalNAc-T3) | 2.7 | 5.05 | Down | NM_004482 |
| GGT6 | Gamma-glutamyltransferase 6 | 2.23 | 3.85 | Down | NM_153338 |
| GNAZ | Guanine nucleotide binding protein (G protein), alpha z polypeptide | 4 | 7.41 | Down | NM_002073 |
| GNG13 | Guanine nucleotide binding protein (G protein), gamma 13 | 57.56 | 22.45 | Down | NM_016541 |
| GPR120 | G protein-coupled receptor 120 | 1.74 | 2.2 | Down | NM_181745 |
| H6PD | Hexose-6-phosphate dehydrogenase (glucose 1-dehydrogenase) | 1.58 | 4.73 | Down | NM_004285 |
| HADH | Hydroxyacyl-Coenzyme A dehydrogenase | 1.6 | 1.54 | Down | NM_005327 |
| hCG_1776259 | Hypothetical protein FLJ23556 | 2.1 | 3.28 | Down | AK027209 |
| HDAC4 | Histone deacetylase 4 | 3.34 | 2.94 | Down | NM_006037 |
| HNRNPA3 | Heterogeneous nuclear ribonucleoprotein A3 | 1.6 | 1.86 | Down | NM_194247 |
| HP1BP3 | Heterochromatin protein 1, binding protein 3 | 1.62 | 1.66 | Down | AK023129 |
| IGLL1 | Immunoglobulin lambda-like polypeptide 1 | 1.65 | 1.62 | Down | NM_020070 |
| ISG15 | ISG15 ubiquitin-like modifier | 1.64 | 1.6 | Down | NM_005101 |
| JAK1 | Janus kinase 1 (a protein tyrosine kinase) | 1.67 | 1.64 | Down | NM_002227 |
| JMJD2C | Jumonji domain containing 2C | 1.78 | 2 | Down | AK098534 |
| KIAA0485 | KIAA0485 protein | 3.06 | 6.06 | Down | AB007954 |
| KIAA1166 | KIAA1166 | 1.59 | 2.28 | Down | AA807922 |
| KIAA1622 | KIAA1622 | 2.43 | 1.53 | Down | NM_058237 |
| KIAA1632 | KIAA1632 | 1.84 | 1.65 | Down | NM_020964 |
| KIF15 | Kinesin family member 15 | 1.56 | 2.55 | Down | NM_020242 |
| KIF17 | Kinesin family member 17 | 1.63 | 2.13 | Down | NM_020816 |
| KIF24 | Kinesin family member 24 | 1.57 | 9.93 | Down | AK001795 |
| KLRC1 | Killer cell lectin-like receptor subfamily C, member 1 | 2.05 | 1.87 | Down | NM_002259 |
| LDB1 | LIM domain binding 1 | 5.29 | 2.68 | Down | NM_003893 |
| LENG8 | Leukocyte receptor cluster (LRC) member 8 | 2.32 | 2.34 | Down | NM_052925 |
| LMBRD2 | LMBR1 domain containing 2 | 1.58 | 2.43 | Down | NM_001007527 |
| LOXL2 | Lysyl oxidase-like 2 | 1.81 | 1.85 | Down | NM_002318 |
| LPA | Lipoprotein, Lp(a) | 1.7 | 2.67 | Down | NM_005577 |
| LPHN1 | Latrophilin 1 | 3.81 | 1.62 | Down | NM_001008701 |
| LRRFIP2 | Leucine rich repeat (in FLII) interacting protein 2 | 1.83 | 1.65 | Down | NM_017724 |
| LSM14A | LSM14A, SCD6 homolog A (S. cerevisiae) | 1.61 | 1.73 | Down | NM_015578 |
| MAGEC1 | Melanoma antigen family C, 1 | 2.93 | 2.85 | Down | NM_005462 |
| MAP1B | Microtubule-associated protein 1B | 1.855 | 1.55 | Down | NM_005909 |
| MASP2 | Mannan-binding lectin serine peptidase 2 | 1.5 | 2.36 | Down | NM_139208 |
| MED26 | Mediator complex subunit 26 | 1.55 | 5.43 | Down | BC030138 |
| METTL7A | Methyltransferase like 7A | 1.66 | 2.67 | Down | NM_014033 |
| MGEA5 | Meningioma expressed antigen 5 (hyaluronidase) | 1.92 | 2.32 | Down | AF307332 |
| MIAT | Myocardial infarction associated transcript (non-protein coding) | 13.33 | 6.04 | Down | AK098753 |
| MKLN1 | Muskelin 1, intracellular mediator containing kelch motifs | 2.01 | 1.9 | Down | NM_013255 |
| MLL3 | Myeloid/lymphoid or mixed-lineage leukemia 3 | 1.89 | 3.05 | Down | NM_170606 |
| MMD | Monocyte to macrophage differentiation-associated | 1.97 | 2.01 | Down | NM_012329 |
| MRPL46 | Mitochondrial ribosomal protein L46 | 1.66 | 1.61 | Down | NM_017996 |
| MSRB2 | Methionine sulfoxide reductase B2 | 1.55 | 1.9 | Down | NM_012228 |
| MUC3A | Mucin 3A, cell surface associated | 3.35 | 2.88 | Down | M55405 |
| MYADM | Myeloid-associated differentiation marker | 1.65 | 1.83 | Down | NM_001020818 |
| MYO1C | Myosin IC | 1.98 | 3.83 | Down | NM_033375 |
| NAIP | NLR family, apoptosis inhibitory protein | 1.93 | 1.7 | Down | NM_004536 |
| NBPF3 | Neuroblastoma breakpoint family, member 3 | 1.55 | 1.77 | Down | NM_032264 |
| NDRG1 | N-myc downstream regulated gene 1 | 2.62 | 1.89 | Down | NM_006096 |
| NEDD4L | Neural precursor cell expressed, developmentally down-regulated 4-like | 3.42 | 1.77 | Down | NM_015277 |
| OGT | O-linked N-acetylglucosamine (GlcNAc) transferase (UDP-N-acetylglucosamine:polypeptide-N-acetylglucosaminyl transferase) | 1.58 | 1.54 | Down | NM_181672 |
| ORAI1 | ORAI calcium release-activated calcium modulator 1 | 1.72 | 4.04 | Down | AK027372 |
| ORAI3 | ORAI calcium release-activated calcium modulator 3 | 1.85 | 2.58 | Down | NM_152288 |
| ORC6L | Origin recognition complex, subunit 6 like (yeast) | 1.56 | 1.62 | Down | NM_014321 |
| OXCT2 | 3-oxoacid CoA transferase 2 | 10.75 | 3.07 | Down | AK024440 |
| PABPC1L | Poly(A) binding protein, cytoplasmic 1-like | 2.05 | 1.64 | Down | BC039151 |
| PAG1 | Phosphoprotein associated with glycosphingolipid microdomains 1 | 2.01 | 2.14 | Down | NM_018440 |
| PAX4 | Paired box 4 | 4.83 | 16.07 | Down | NM_006193 |
| PCMTD2 | Protein-L-isoaspartate (D-aspartate) O-methyltransferase domain containing 2 | 1.92 | 2.19 | Down | BC032332 |
| PCSK4 | Proprotein convertase subtilisin/kexin type 4 | 1.5 | 2.78 | Down | NM_017573 |
| PDE7A | Phosphodiesterase 7A | 1.58 | 4.37 | Down | NM_002604 |
| PDZD7 | PDZ domain containing 7 | 2.34 | 4.6 | Down | NM_024895 |
| PHF20 | PHD finger protein 20 | 1.52 | 2.33 | Down | AK090798 |
| PHF20L1 | PHD finger protein 20-like 1 | 2.41 | 3.28 | Down | NM_032205 |
| POLR2J2 | Uroplakin-like protein | 2.05 | 2.31 | Down | NM_032959 |
| POM121 | POM121 membrane glycoprotein (rat) | 1.57 | 3.36 | Down | NM_172020 |
| POU5F1 | POU class 5 homeobox 1 | 2.41 | 2.5 | Down | NM_002701 |
| PPP1R16B | Protein phosphatase 1, regulatory (inhibitor) subunit 16B | 1.61 | 1.69 | Down | NM_015568 |
| PPP3CA | Protein phosphatase 3 (formerly 2B), catalytic subunit, alpha isoform | 1.71 | 1.7 | Down | BU618279 |
| PQLC3 | PQ loop repeat containing 3 | 1.55 | 1.89 | Down | NM_152391 |
| PRDX3 | Peroxiredoxin 3 | 1.66 | 3.14 | Down | NM_006793 |
| PRKCH | Protein kinase C, eta | 3.54 | 1.85 | Down | NM_006255 |
| PRO0478 | PRO0478 protein | 2.27 | 3.97 | Down | AF090930 |
| PRR11 | Proline rich 11 | 2.31 | 1.65 | Down | AK001891 |
| PRRT2 | Proline-rich transmembrane protein 2 | 3.18 | 2.89 | Down | NM_145239 |
| PRX | Periaxin | 1.84 | 1.55 | Down | NM_020956 |
| PTGR2 | Prostaglandin reductase 2 | 2.56 | 1.64 | Down | NM_152444 |
| PXN | Paxillin | 2.31 | 4.53 | Down | BC052611 |
| RAB37 | RAB37, member RAS oncogene family | 4.13 | 2.72 | Down | NM_175738 |
| RAX2 | Retina and anterior neural fold homeobox 2 | 2.55 | 4.29 | Down | NM_032753 |
| RBM17 | RNA binding motif protein 17 | 1.67 | 1.95 | Down | NM_032905 |
| RGS11 | Regulator of G-protein signaling 11 | 1.89 | 3.57 | Down | NM_003834 |
| RICTOR | Rapamycin-insensitive companion of mTOR | 1.66 | 1.6 | Down | NM_152756 |
| RNF213 | Ring finger protein 213 | 1.53 | 1.98 | Down | NM_020914 |
| RPA4 | Replication protein A4, 34kDa | 2.07 | 2.56 | Down | NM_013347 |
| RPL28 | Ribosomal protein L28 | 2.34 | 2.15 | Down | NM_000991 |
| RPL29 | Ribosomal protein L29 | 1.79 | 1.71 | Down | NM_000992 |
| RUNDC2B | RUN domain containing 2B | 1.94 | 2.87 | Down | AK023827 |
| S100PBP | S100P binding protein | 1.93 | 1.98 | Down | NM_022753 |
| SELPLG | Selectin P ligand | 1.63 | 4.35 | Down | NM_003006 |
| SERPINB9 | Serpin peptidase inhibitor, clade B (ovalbumin), member 9 | 2.43 | 2.68 | Down | NM_004155 |
| SETD5 | SET domain containing 5 | 1.97 | 4.01 | Down | BX648380 |
| SLC26A6 | Solute carrier family 26, member 6 | 1.7 | 2.4 | Down | AF161369 |
| SLC27A1 | Solute carrier family 27 (fatty acid transporter), member 1 | 2.49 | 2.31 | Down | NM_198580 |
| SLC35E4 | Solute carrier family 35, member E4 | 1.81 | 4.36 | Down | BC031973 |
| SLC7A11 | Solute carrier family 7, (cationic amino acid transporter, y+ system) member 11 | 1.93 | 3.66 | Down | NM_014331 |
| SLC9A5 | Solute carrier family 9 (sodium/hydrogen exchanger), member 5 | 1.84 | 1.54 | Down | NM_004594 |
| SMA4 | Glucuronidase, beta pseudogene | 1.82 | 6.86 | Down | L40520 |
| SMC1A | Structural maintenance of chromosomes 1A | 2.32 | 1.88 | Down | NM_006306 |
| SMCR5 | Smith-Magenis syndrome chromosome region, candidate 5 | 2.61 | 2.66 | Down | AF467442 |
| SORL1 | Sortilin-related receptor, L(DLR class) A repeats-containing | 1.74 | 1.86 | Down | NM_003105 |
| SOX12 | SRY (sex determining region Y)-box 12 | 1.51 | 1.95 | Down | NM_006943 |
| SOX4 | SRY (sex determining region Y)-box 4 | 2.36 | 1.7 | Down | AW946823 |
| SS18 | Synovial sarcoma translocation, chromosome 18 | 1.61 | 1.56 | Down | NM_001007559 |
| STAG3L1 | Stromal antigen 3-like 1 | 2.44 | 3.18 | Down | AL137492 |
| STARD3NL | STARD3 N-terminal like | 1.6 | 2.07 | Down | NM_032016 |
| STAU1 | Staufen, RNA binding protein, homolog 1 (Drosophila) | 1.64 | 2.76 | Down | NM_017453 |
| STRBP | Chromosome 9 open reading frame 45 | 2.85 | 2.22 | Down | AK025613 |
| STX16 | Syntaxin 16 | 1.57 | 1.61 | Down | NM_001001433 |
| TACR2 | Tachykinin receptor 2 | 3.01 | 4.79 | Down | NM_001057 |
| TAS2R10 | Taste receptor, type 2, member 10 | 4.22 | 1.52 | Down | NM_023921 |
| TBC1D1 | TBC1 (tre-2/USP6, BUB2, cdc16) domain family, member 1 | 2.44 | 1.57 | Down | NM_015173 |
| tcag7.1017 | Similar to Williams Beuren syndrome chromosome region 19 | 1.67 | 2.37 | Down | BC100972 |
| tcag7.907 | Hypothetical LOC402483 | 1.84 | 2.42 | Down | AK093729 |
| TCFL5 | Transcription factor-like 5 (basic helix-loop-helix) | 1.54 | 2.29 | Down | BC065520 |
| TCL1B | T-cell leukemia/lymphoma 1B | 2.33 | 1.81 | Down | NM_199206 |
| TDRD10 | Tudor domain containing 10 | 67.51 | 37.36 | Down | NM_182499 |
| TGFBR2 | Transforming growth factor, beta receptor II (70/80kDa) | 1.7 | 1.56 | Down | NM_001024847 |
| TMCC2 | Transmembrane and coiled-coil domain family 2 | 51 | 19.38 | Down | NM_014858 |
| TNFRSF25 | Tumor necrosis factor receptor superfamily, member 25 | 2.57 | 1.75 | Down | NM_148965 |
| TNRC6B | Trinucleotide repeat containing 6B | 2.26 | 2.55 | Down | BF960555 |
| TOP2B | Topoisomerase (DNA) II beta 180kDa | 1.58 | 2.37 | Down | NM_001068 |
| TRAF5 | TNF receptor-associated factor 5 | 2.11 | 1.54 | Down | NM_004619 |
| TRIM52 | Tripartite motif-containing 52 | 1.55 | 2.96 | Down | NM_032765 |
| TRIM74 | Tripartite motif-containing 74 | 3.96 | 4.33 | Down | NM_198853 |
| TRIOBP | TRIO and F-actin binding protein | 1.8 | 1.51 | Down | NM_001039141 |
| TTC21A | Tetratricopeptide repeat domain 21A | 2.81 | 2.5 | Down | NM_145755 |
| TTC25 | Tetratricopeptide repeat domain 25 | 2.11 | 2.26 | Down | NM_031421 |
| TUBB3 | Tubulin, beta 3 | 2.56 | 2.71 | Down | NM_006086 |
| UBE2E2 | Ubiquitin-conjugating enzyme E2E 2 (UBC4/5 homolog, yeast) | 1.81 | 1.5 | Down | NM_152653 |
| UBE3A | Ubiquitin protein ligase E3A (human papilloma virus E6-associated protein, Angelman syndrome) | 1.62 | 1.88 | Down | AF037219 |
| UBOX5 | U-box domain containing 5 | 2.47 | 4.57 | Down | NM_014948 |
| UBR2 | Ubiquitin protein ligase E3 component n-recognin 2 | 2.28 | 4.6 | Down | AK001118 |
| UCHL1 | Ubiquitin carboxyl-terminal esterase L1 (ubiquitin thiolesterase) | 3.77 | 3.72 | Down | NM_004181 |
| UPK2 | Uroplakin 2 | 2.9 | 1.71 | Down | NM_006760 |
| USP47 | Ubiquitin specific peptidase 47 | 1.83 | 1.68 | Down | NM_017944 |
| VPS13C | Vacuolar protein sorting 13 homolog C (S. cerevisiae) | 1.97 | 4.36 | Down | AK056744 |
| WBSCR19 | Williams Beuren syndrome chromosome region 19 | 2.51 | 2.46 | Down | NM_175064 |
| WHSC1 | Wolf-Hirschhorn syndrome candidate 1 | 1.81 | 2.23 | Down | NM_133334 |
| YPEL1 | Yippee-like 1 (Drosophila) | 1.82 | 2.07 | Down | NM_013313 |
| ZBTB20 | Zinc finger and BTB domain containing 20 | 2.72 | 4.68 | Down | BC010934 |
| ZC3H11A | Zinc finger CCCH-type containing 11A | 1.55 | 1.52 | Down | NM_014827 |
| ZDHHC21 | Zinc finger, DHHC-type containing 21 | 1.78 | 1.93 | Down | NM_178566 |
| ZFP62 | Zinc finger protein 62 homolog (mouse) | 1.61 | 1.64 | Down | AK091550 |
| ZKSCAN1 | Zinc finger with KRAB and SCAN domains 1 | 2.12 | 3.26 | Down | AY260738 |
| ZNF2 | Zinc finger protein 2 | 2.81 | 3.31 | Down | NM_021088 |
| ZNF362 | Zinc finger protein 362 | 1.63 | 1.62 | Down | AK021842 |
| ZNF521 | Zinc finger protein 521 | 6.82 | 2.15 | Down | NM_015461 |
| ZNF713 | Zinc finger protein 713 | 2.74 | 3.88 | Down | NM_182633 |
| ZNF786 | Zinc finger protein 786 | 2.57 | 1.56 | Down | NM_152411 |
| 9-Sep | Septin 9 | 1.65 | 1.55 | Up | NM_006640 |
| ACTG1 | Actin, gamma 1 | 1.76 | 1.62 | Up | NM_001614 |
| AGMAT | Agmatine ureohydrolase (agmatinase) | 1.83 | 1.99 | Up | NM_024758 |
| AMDHD2 | Amidohydrolase domain containing 2 | 2.39 | 1.97 | Up | NM_015944 |
| AMMECR1 | Alport syndrome, mental retardation, midface hypoplasia and elliptocytosis chromosomal region, gene 1 | 1.58 | 1.99 | Up | NM_015365 |
| AP3M2 | Adaptor-related protein complex 3, mu 2 subunit | 1.71 | 1.61 | Up | NM_006803 |
| APOBEC3G | Apolipoprotein B mRNA editing enzyme, catalytic polypeptide-like 3G | 2.06 | 2.37 | Up | NM_021822 |
| APOBEC3H | Apolipoprotein B mRNA editing enzyme, catalytic polypeptide-like 3H | 1.98 | 4.69 | Up | NM_181773 |
| APOOL | Apolipoprotein O-like | 1.74 | 2.31 | Up | NM_198450 |
| ARFGEF2 | ADP-ribosylation factor guanine nucleotide-exchange factor 2 (brefeldin A-inhibited) | 1.71 | 1.86 | Up | NM_006420 |
| ARHGAP19 | Rho GTPase activating protein 19 | 1.82 | 1.53 | Up | NM_032900 |
| ARP11 | Actin-related Arp11 | 2.3 | 2.7 | Up | AB039791 |
| ATMIN | ATM interactor | 1.64 | 1.88 | Up | NM_015251 |
| AVIL | Advillin | 2.15 | 1.63 | Up | BX647344 |
| B3GALNT2 | Beta-1,3-N-acetylgalactosaminyltransferase 2 | 1.6 | 1.77 | Up | NM_152490 |
| BAX | BCL2-associated X protein | 2.09 | 1.73 | Up | NM_138763 |
| BBS9 | Bardet-Biedl syndrome 9 | 1.94 | 1.67 | Up | BC032715 |
| BCCIP | BRCA2 and CDKN1A interacting protein | 1.79 | 1.98 | Up | NM_078469 |
| BCL2L12 | BCL2-like 12 (proline rich) | 1.81 | 1.66 | Up | AA856716 |
| BCLAF1 | BCL2-associated transcription factor 1 | 1.55 | 1.6 | Up | NM_014739 |
| BICD2 | Bicaudal D homolog 2 (Drosophila) | 2.1 | 2.02 | Up | NM_015250 |
| BIK | BCL2-interacting killer (apoptosis-inducing) | 1.59 | 1.71 | Up | NM_001197 |
| CALR | Calreticulin | 2.06 | 1.65 | Up | CA306742 |
| CAMKK2 | Calcium/calmodulin-dependent protein kinase kinase 2, beta | 2.65 | 1.68 | Up |  |
| CAPRIN2 | Caprin family member 2 | 1.51 | 1.72 | Up | NM_001002259 |
| CARD6 | Caspase recruitment domain family, member 6 | 2.38 | 5.01 | Up | NM_032587 |
| CARD9 | Caspase recruitment domain family, member 9 | 1.71 | 2.74 | Up | NM_052813 |
| CASP10 | Caspase 10, apoptosis-related cysteine peptidase | 2.78 | 1.7 | Up | NM_032977 |
| CATSPER3 | Cation channel, sperm associated 3 | 1.67 | 1.78 | Up | NM_178019 |
| CCNK | Cyclin K | 1.55 | 1.74 | Up | NM_003858 |
| CCT5 | Chaperonin containing TCP1, subunit 5 (epsilon) | 1.67 | 1.7 | Up | NM_012073 |
| CD300A | CD300a molecule | 1.55 | 1.73 | Up | NM_007261 |
| CD52 | CD52 molecule | 1.73 | 2.88 | Up | NM_001803 |
| CD80 | CD80 molecule | 1.65 | 2 | Up | NM_005191 |
| CDC40 | Cell division cycle 40 homolog (S. cerevisiae) | 1.58 | 1.63 | Up |  |
| CDKN1A | Cyclin-dependent kinase inhibitor 1A (p21, Cip1) | 1.58 | 2.57 | Up | NM_078467 |
| CHST11 | Carbohydrate (chondroitin 4) sulfotransferase 11 | 3.29 | 1.5 | Up | NM_018413 |
| CNPY2 | Canopy 2 homolog (zebrafish) | 1.59 | 1.71 | Up | BC001027 |
| CNTF | ZFP91-CNTF | 1.61 | 1.52 | Up | NM_170768 |
| COG8 | Peptide deformylase (mitochondrial) | 2.71 | 1.53 | Up | NM_032382 |
| COL6A1 | Collagen, type VI, alpha 1 | 1.65 | 1.68 | Up | NM_001848 |
| COMMD1 | Copper metabolism (Murr1) domain containing 1 | 1.58 | 1.96 | Up | NM_152516 |
| CORO1C | Coronin, actin binding protein, 1C | 1.66 | 1.88 | Up | NM_014325 |
| CUGBP1 | CUG triplet repeat, RNA binding protein 1 | 1.84 | 2.28 | Up | NM_198700 |
| CYB5D1 | Cytochrome b5 domain containing 1 | 2.54 | 2.11 | Up | NM_144607 |
| DDX31 | DEAD (Asp-Glu-Ala-Asp) box polypeptide 31 | 2.6 | 1.77 | Up | NM_138620 |
| DDX42 | DEAD (Asp-Glu-Ala-Asp) box polypeptide 42 | 1.75 | 2.18 | Up | NM_007372 |
| DEGS1 | Degenerative spermatocyte homolog 1, lipid desaturase (Drosophila) | 1.53 | 1.91 | Up | NM_003676 |
| DGKE | Diacylglycerol kinase, epsilon 64kDa | 2.01 | 2 | Up | NM_003647 |
| DHX33 | DEAH (Asp-Glu-Ala-His) box polypeptide 33 | 2.73 | 2.08 | Up | NM_020162 |
| DIP2B | DIP2 disco-interacting protein 2 homolog B (Drosophila) | 1.67 | 1.78 | Up | NM_173602 |
| DPP9 | Dipeptidyl-peptidase 9 | 2.24 | 2.28 | Up | NM_139159 |
| DUSP2 | Dual specificity phosphatase 2 | 2.2 | 2.43 | Up | NM_004418 |
| DUSP26 | Dual specificity phosphatase 26 (putative) | 1.73 | 4.33 | Up | NM_024025 |
| DYNC1LI2 | Dynein, cytoplasmic 1, light intermediate chain 2 | 1.52 | 1.69 | Up | NM_006141 |
| EGR1 | Early growth response 1 | 6.04 | 12.16 | Up | NM_001964 |
| ENTPD7 | Ectonucleoside triphosphate diphosphohydrolase 7 | 1.61 | 1.86 | Up | NM_020354 |
| EPB41 | Erythrocyte membrane protein band 4.1 (elliptocytosis 1, RH-linked) | 1.61 | 2.38 | Up | NM_203342 |
| ESF1 | ESF1, nucleolar pre-rRNA processing protein, homolog (S. cerevisiae) | 1.64 | 1.87 | Up | NM_016649 |
| FASTKD2 | FAST kinase domains 2 | 1.57 | 1.67 | Up | NM_014929 |
| FBP1 | Fructose-1,6-bisphosphatase 1 | 6.18 | 1.58 | Up | NM_000507 |
| FBXO6 | F-box protein 6 | 2.32 | 1.92 | Up | NM_018438 |
| FLVCR1 | Feline leukemia virus subgroup C cellular receptor 1 | 2.11 | 2.3 | Up | NM_014053 |
| FNTB | Farnesyltransferase, CAAX box, beta | 2.02 | 1.51 | Up | NM_002028 |
| FOXC1 | Forkhead box C1 | 2.36 | 1.51 | Up | NM_001453 |
| GDAP1 | Ganglioside-induced differentiation-associated protein 1 | 2.51 | 2.32 | Up | NM_018972 |
| GEMIN6 | Gem (nuclear organelle) associated protein 6 | 2.18 | 1.85 | Up | NM_024775 |
| GGCX | Gamma-glutamyl carboxylase | 1.92 | 1.5 | Up | NM_000821 |
| GOSR2 | Golgi SNAP receptor complex member 2 | 1.54 | 1.65 | Up | NM_054022 |
| GPATCH4 | G patch domain containing 4 | 1.5 | 1.62 | Up | NM_182679 |
| GPR146 | G protein-coupled receptor 146 | 2.45 | 1.66 | Up | NM_138445 |
| GPSM3 | G-protein signaling modulator 3 (AGS3-like, C. elegans) | 2.22 | 1.52 | Up | NM_022107 |
| HDLBP | High density lipoprotein binding protein (vigilin) | 1.58 | 1.8 | Up | AF116718 |
| HEATR3 | HEAT repeat containing 3 | 2.28 | 2.45 | Up | NM_182922 |
| HERC2 | Hect domain and RLD 2 | 1.85 | 1.53 | Up | NM_004667 |
| HIST2H2AB | Histone cluster 2, H2ab | 2.79 | 1.72 | Up | NM_175065 |
| HLA-DOA | Major histocompatibility complex, class II, DO alpha | 1.76 | 1.7 | Up | NM_002119 |
| HLA-DPA1 | Major histocompatibility complex, class II, DP alpha 1 | 1.82 | 3.63 | Up | NM_033554 |
| HLA-DPB1 | Major histocompatibility complex, class II, DP beta 1 | 1.56 | 1.6 | Up | NM_002121 |
| HOOK3 | Hook homolog 3 (Drosophila) | 1.62 | 2.06 | Up | NM_032410 |
| HUWE1 | HECT, UBA and WWE domain containing 1 | 2.27 | 1.52 | Up | NM_031407 |
| INF2 | Inverted formin, FH2 and WH2 domain containing | 1.92 | 1.51 | Up | NM_032714 |
| ING5 | Inhibitor of growth family, member 5 | 2.03 | 1.62 | Up | NM_032329 |
| INTS5 | Integrator complex subunit 5 | 1.79 | 1.54 | Up | NM_030628 |
| ITGA4 | Integrin, alpha 4 (antigen CD49D, alpha 4 subunit of VLA-4 receptor) | 1.56 | 1.94 | Up | NM_000885 |
| KIAA0241 | KIAA0241 | 1.58 | 1.72 | Up | NM_015060 |
| KIAA1967 | KIAA1967 | 1.84 | 1.54 | Up | NM_021174 |
| KLHDC5 | Kelch domain containing 5 | 1.59 | 2.02 | Up | NM_020782 |
| LIG3 | Ligase III, DNA, ATP-dependent | 1.8 | 1.94 | Up | NM_002311 |
| LIX1L | Lix1 homolog (mouse)-like | 2.39 | 1.72 | Up | NM_153713 |
| LONP2 | Lon peptidase 2, peroxisomal | 1.8 | 1.71 | Up | NM_031490 |
| LPGAT1 | Lysophosphatidylglycerol acyltransferase 1 | 1.6 | 1.78 | Up | NM_014873 |
| MAN1A2 | Mannosidase, alpha, class 1A, member 2 | 1.63 | 2.25 | Up | NM_006699 |
| MBD1 | Methyl-CpG binding domain protein 1 | 1.5 | 1.73 | Up | NM_015845 |
| MBOAT2 | Membrane bound O-acyltransferase domain containing 2 | 10.79 | 1.7 | Up | NM_138799 |
| MCCC2 | Methylcrotonoyl-Coenzyme A carboxylase 2 (beta) | 1.55 | 1.56 | Up | NM_022132 |
| METTL1 | Methyltransferase like 1 | 2.16 | 1.63 | Up | NM_005371 |
| MFSD9 | Major facilitator superfamily domain containing 9 | 1.9 | 1.53 | Up | NM_032718 |
| MLKL | Mixed lineage kinase domain-like | 1.72 | 2.02 | Up | NM_152649 |
| MLX | MAX-like protein X | 1.51 | 1.81 | Up | NM_170607 |
| MPZL1 | Myelin protein zero-like 1 | 1.52 | 2.45 | Up | NM_003953 |
| MREG | Melanoregulin | 2.31 | 2.04 | Up | NM_018000 |
| MRPS10 | Mitochondrial ribosomal protein S10 | 1.91 | 1.64 | Up | NM_018141 |
| MXD1 | MAX dimerization protein 1 | 2.45 | 2.6 | Up | NM_002357 |
| N6AMT2 | N-6 adenine-specific DNA methyltransferase 2 (putative) | 1.56 | 1.68 | Up | NM_174928 |
| NAPG | N-ethylmaleimide-sensitive factor attachment protein, gamma | 1.55 | 2.46 | Up | NM_003826 |
| NFATC2IP | Nuclear factor of activated T-cells, cytoplasmic, calcineurin-dependent 2 interacting protein | 1.97 | 1.55 | Up | NM_032815 |
| NIPA1 | Non imprinted in Prader-Willi/Angelman syndrome 1 | 1.6 | 2.06 | Up | NM_144599 |
| NLN | Neurolysin (metallopeptidase M3 family) | 1.74 | 1.65 | Up | NM_020726 |
| NOL10 | Nucleolar protein 10 | 1.73 | 1.92 | Up | NM_024894 |
| NOLC1 | Nucleolar and coiled-body phosphoprotein 1 | 1.63 | 1.52 | Up | NM_004741 |
| N-PAC | Cytokine-like nuclear factor n-pac | 1.66 | 1.77 | Up | NM_032569 |
| NUDT16L1 | Nudix (nucleoside diphosphate linked moiety X)-type motif 16-like 1 | 1.89 | 2.11 | Up | NM_032349 |
| OTUB1 | OTU domain, ubiquitin aldehyde binding 1 | 1.51 | 1.59 | Up | NM_017670 |
| OTUD4 | OTU domain containing 4 | 1.67 | 2.48 | Up | NM_199324 |
| OTUD7A | OTU domain containing 7A | 2.7 | 1.65 | Up | NM_130901 |
| PANK3 | Pantothenate kinase 3 | 1.61 | 1.56 | Up | NM_024594 |
| PGAM5 | Phosphoglycerate mutase family member 5 | 1.98 | 1.56 | Up | NM_138575 |
| PHF15 | PHD finger protein 15 | 1.55 | 2.73 | Up | NM_015288 |
| PIGR | Polymeric immunoglobulin receptor | 2.71 | 1.61 | Up | NM_002644 |
| PIP4K2B | Phosphatidylinositol-5-phosphate 4-kinase, type II, beta | 2.15 | 1.92 | Up | NM_003559 |
| PLEKHB2 | Pleckstrin homology domain containing, family B (evectins) member 2 | 1.82 | 2.16 | Up | NM_017958 |
| PML | Promyelocytic leukemia | 1.83 | 1.75 | Up | NM_033247 |
| POLR2L | Polymerase (RNA) II (DNA directed) polypeptide L, 7.6kDa | 1.74 | 2.15 | Up | NM_021128 |
| POP1 | Processing of precursor 1, ribonuclease P/MRP subunit (S. cerevisiae) | 2.77 | 2.19 | Up | NM_015029 |
| PPM1H | Protein phosphatase 1H (PP2C domain containing) | 3.21 | 1.52 | Up | AB032983 |
| PPM1K | Protein phosphatase 1K (PP2C domain containing) | 1.52 | 1.87 | Up | BC041350 |
| PPME1 | Protein phosphatase methylesterase 1 | 1.77 | 1.88 | Up | NM_016147 |
| PRKAR1B | Protein kinase, cAMP-dependent, regulatory, type I, beta | 2.44 | 2.19 | Up | NM_002735 |
| PTPN6 | Protein tyrosine phosphatase, non-receptor type 6 | 8.37 | 2.32 | Up | NM_002831 |
| PTPRJ | Protein tyrosine phosphatase, receptor type, J | 2.48 | 2.52 | Up | NM_002843 |
| RAB27A | RAB27A, member RAS oncogene family | 1.61 | 2.2 | Up | NM_004580 |
| RABGAP1L | RAB GTPase activating protein 1-like | 1.68 | 2.02 | Up | NM_014857 |
| RBBP5 | Retinoblastoma binding protein 5 | 1.75 | 2 | Up | NM_005057 |
| RBM47 | RNA binding motif protein 47 | 2.14 | 2.9 | Up | NM_019027 |
| RERE | Arginine-glutamic acid dipeptide (RE) repeats | 1.51 | 1.95 | Up | NM_012102 |
| RGP1 | RGP1 retrograde golgi transport homolog (S. cerevisiae) | 2.25 | 2.82 | Up | BC001725 |
| RGS16 | Regulator of G-protein signaling 16 | 2.2 | 1.74 | Up | NM_002928 |
| RNMT | RNA (guanine-7-) methyltransferase | 1.69 | 2.24 | Up | NM_003799 |
| RP5-1000E10.4 | Suppressor of IKK epsilon | 1.62 | 1.97 | Up | NM_025073 |
| RPS26 | Ribosomal protein S26 | 2.27 | 2.46 | Up | NM_001029 |
| RRAGD | Ras-related GTP binding D | 1.53 | 1.65 | Up | NM_021244 |
| SCD5 | Stearoyl-CoA desaturase 5 | 1.96 | 2.74 | Up | NM_001037582 |
| SECTM1 | Secreted and transmembrane 1 | 5.62 | 37.89 | Up | NM_003004 |
| SEH1L | SEH1-like (S. cerevisiae) | 1.58 | 1.78 | Up | NM_031216 |
| SETD8 | SET domain containing (lysine methyltransferase) 8 | 1.51 | 1.99 | Up | NM_020382 |
| SFRS4 | Splicing factor, arginine/serine-rich 4 | 1.66 | 1.54 | Up | NM_005626 |
| SGPL1 | Sphingosine-1-phosphate lyase 1 | 2.19 | 2.08 | Up | NM_003901 |
| SGPP2 | Sphingosine-1-phosphate phosphotase 2 | 3.25 | 1.51 | Up | NM_152386 |
| SH3BP2 | SH3-domain binding protein 2 | 2.12 | 2.1 | Up | NM_003023 |
| SIAH2 | Seven in absentia homolog 2 (Drosophila) | 1.71 | 1.52 | Up | NM_005067 |
| SLC16A7 | Solute carrier family 16, member 7 (monocarboxylic acid transporter 2) | 1.95 | 3.84 | Up | NM_004731 |
| SLC29A3 | Solute carrier family 29 (nucleoside transporters), member 3 | 1.74 | 1.9 | Up | NM_018344 |
| SLC35D1 | Solute carrier family 35 (UDP-glucuronic acid/UDP-N-acetylgalactosamine dual transporter), member D1 | 2.15 | 2.2 | Up | NM_015139 |
| SLC39A9 | Solute carrier family 39 (zinc transporter), member 9 | 1.53 | 2.13 | Up | NM_018375 |
| SMCR7L | Smith-Magenis syndrome chromosome region, candidate 7-like | 1.72 | 1.57 | Up | NM_019008 |
| SOCS7 | Suppressor of cytokine signaling 7 | 1.81 | 2.16 | Up | NM_014598 |
| SOLH | Small optic lobes homolog (Drosophila) | 2.74 | 2.03 | Up | BC032648 |
| SURF4 | Surfeit 4 | 1.63 | 1.63 | Up | NM_033161 |
| TCP11L2 | T-complex 11 (mouse)-like 2 | 1.76 | 1.82 | Up | NM_152772 |
| TDG | Thymine-DNA glycosylase | 1.59 | 1.82 | Up | NM_003211 |
| TDH | L-threonine dehydrogenase | 7.37 | 2.21 | Up | BC080558 |
| TEX261 | Testis expressed 261 | 1.5 | 1.5 | Up | NM_144582 |
| THRAP3 | Thyroid hormone receptor associated protein 3 | 1.7 | 1.83 | Up | NM_005119 |
| TIMM8A | Translocase of inner mitochondrial membrane 8 homolog A (yeast) | 1.51 | 1.73 | Up | NM_004085 |
| TMEM102 | Transmembrane protein 102 | 1.57 | 1.58 | Up | NM_178518 |
| TMEM2 | Transmembrane protein 2 | 2.16 | 1.75 | Up | NM_013390 |
| TMEM33 | Transmembrane protein 33 | 1.74 | 1.78 | Up | NM_018126 |
| TNF | Tumor necrosis factor (TNF superfamily, member 2) | 2.74 | 1.56 | Up | NM_000594 |
| TNFRSF10C | Tumor necrosis factor receptor superfamily, member 10c, decoy without an intracellular domain | 1.94 | 1.66 | Up | BC021569 |
| TNFSF13 | Tumor necrosis factor (ligand) superfamily, member 12 | 1.63 | 2.23 | Up | NM_003809 |
| TOLLIP | Toll interacting protein | 1.61 | 1.6 | Up | NM_019009 |
| TOR1AIP1 | Torsin A interacting protein 1 | 1.67 | 1.72 | Up | NM_015602 |
| TP53I3 | Tumor protein p53 inducible protein 3 | 2.14 | 2.65 | Up | NM_004881 |
| TPCN1 | Two pore segment channel 1 | 1.5 | 2.26 | Up | AB032995 |
| TPM4 | Tropomyosin 4 | 1.55 | 1.7 | Up | NM_003290 |
| TRIM5 | Tripartite motif-containing 5 | 1.68 | 2.86 | Up | NM_033092 |
| TTC26 | Tetratricopeptide repeat domain 26 | 2.7 | 1.58 | Up | NM_024926 |
| UBAP2L | Ubiquitin associated protein 2-like | 1.6 | 2.56 | Up | NM_014847 |
| UBE2G2 | Ubiquitin-conjugating enzyme E2G 2 (UBC7 homolog, yeast) | 1.68 | 1.66 | Up | NM_182688 |
| UBE3C | Ubiquitin protein ligase E3C | 1.84 | 1.94 | Up | NM_014671 |
| UFM1 | Ubiquitin-fold modifier 1 | 1.54 | 2.05 | Up | NM_016617 |
| UGCG | UDP-glucose ceramide glucosyltransferase | 2.06 | 2.32 | Up | NM_003358 |
| UTP14A | UTP14, U3 small nucleolar ribonucleoprotein, homolog A (yeast) | 1.6 | 1.84 | Up | NM_006649 |
| VAMP5 | Vesicle-associated membrane protein 5 (myobrevin) | 2.1 | 1.63 | Up | NM_006634 |
| VCPIP1 | Valosin containing protein (p97)/p47 complex interacting protein 1 | 1.55 | 1.69 | Up | NM_025054 |
| WDR33 | WD repeat domain 33 | 1.77 | 1.61 | Up | NM_018383 |
| WDR36 | WD repeat domain 36 | 1.94 | 1.74 | Up | NM_139281 |
| XPO4 | Exportin 4 | 1.66 | 1.78 | Up | NM_022459 |
| YIPF5 | Yip1 domain family, member 5 | 1.66 | 1.62 | Up | NM_030799 |
| ZBTB24 | Zinc finger and BTB domain containing 24 | 1.67 | 2.19 | Up | NM_014797 |
| ZBTB38 | Zinc finger and BTB domain containing 38 | 1.87 | 1.53 | Up | AK094201 |
| ZCCHC2 | Zinc finger, CCHC domain containing 2 | 2.42 | 1.52 | Up | NM_017742 |
| ZNF276 | Zinc finger protein 276 | 2.14 | 1.58 | Up | NM_152287 |
| ZNF485 | Zinc finger protein 485 | 1.56 | 1.96 | Up | NM_145312 |
| ZNF518B | Zinc finger protein 518B | 2.02 | 1.83 | Up | NM_053042 |
| ZNF552 | Zinc finger protein 552 | 1.64 | 1.67 | Up | NM_024762 |
| ZNF576 | Zinc finger protein 576 | 1.73 | 1.74 | Up | NM_024327 |
| ZNHIT2 | Zinc finger, HIT type 2 | 1.57 | 1.55 | Up | NM_014205 |
