# Supplementary material for: Expression and Functional Studies of Ubiquitin C-Terminal Hydrolase L1 Regulated Genes
Source: PLoS One. 2009 Aug 26;4(8):e6764. doi: 10.1371/journal.pone.0006764 (PMC2729380; doi:10.1371/journal.pone.0006764)
Supplement: Table S2 — List of QRT-PCR primers (0.05 MB DOC) [file pone.0006764.s005.doc]

| BIK Forward | ACCTGGACCCTATGGAGGAC |
| --- | --- |
| BIK Reverse | GGTGAAACCGTCCATGAAAC |
| BAX Forward | GCTGGACATTGGACTTCCTC |
| BAX Reverse | CCTCCCAGAAAAATGCCATA |
| MYC Forward | TTCGGGTAGTGGAAAACCAG |
| MYC Reverse | CAGCAGCTCGAATTTCTTCC |
| CDKN1A (p21) Forward | TTAGCAGCGGAACAAGGAGT |
| CDKN1A (p21) Reverse | GCCGAGAGAAAACAGTCCAG |
| PXN Forward | aactggttgaagggtgttgc |
| PXN Reverse | caccagctttcctgagaagg |
| FASTKD2 Forward | TGTGGACAATTGCCAAAAGA |
| FASTKD2 Reverse | CAGCAAAGTCTGCACCAAAA |
| BIRC6 Forward | GGCTCATAATGCAGTGCAGA |
| BIRC6 Reverse | CTGCAGGAACAGAGTGTCCA |
| APC Forward | AAGAAGCTCTGCTGCCCATA |
| APC Reverse | TAGGTCGGCTGGGTATTGAC |
| SIAH2 Forward | CTGTCGACTGGGTGATGATG |
| SIAH2 Reverse | CACACCGTCATGAATCGAAC |
| JAK1 Forward | AGCGATGTCCTTACCACACC |
| JAK1 Reverse | CCTCAACACACTCAGGAGCA |
| MUC3A Forward | CAACCAGCTCCTCTCTGACC |
| MUC3A Reverse | GGAGGTAGGACAGGTGACCA |
| RHOA Forward | AAGGACCAGTTCCCAGAGGT |
| RHOA Reverse | TTCTGGGGTCCACTTTTCTG |
| UCH L1 Forward | GGATGGCCACCTCTATGAAC |
| UCH L1 Reverse | AGACCTTGGCAGCGTCCT |
| GAPDH Forward | AGGTGAAGGTCGGAGTCAACG |
| GAPDH Reverse | AGGGGTCATTGATGGCAACA |
| FN1 Forward | ACCAACCTACGGATGACTCG |
| FN1 Reverse | GCTCATCATCTGGCCATTTT |
| Twist2 Forward | CAGAGCCTTTCCAGCAACTC |
| Twist2 Reverse | TCGCTCGACTTCTTGCTGTA |
| LFA1 Forward | CAAGCTGGCTGAAAACAACA |
| LFA1 Reverse | ATTGCTGCAGAAGGAGTCGT |
| MKI67 Forward | AAGCCCTCCAGCTCCTAGTC |
| MKI67 Reverse | TCCGAAGCACCACTTCTTCT |
| TCF4 Forward | GCAGAGTCTCCTTGGAGGTG |
| TCF4 Reverse | GTGCTTGCTGATGGAGCATA |
| LEF1 Forward | AACATGGTGGAAAACGAAGC |
| LEF1 Reverse | GGGTTGGCAGTGATTGTCTT |
| ISG15 Forward | TGTCGGTGTCAGAGCTGAAG |
| ISG15 Reverse | GCCCTTGTTATTCCTCACCA |
